# Inferring homologous protein-protein interactions through pair position specific scoring matrix

**DOI:** 10.1186/1471-2105-14-S2-S11

**Published:** 2013-01-21

**Authors:** Chun-Yu Lin, Yung-Chiang Chen, Yu-Shu Lo, Jinn-Moon Yang

**Affiliations:** 1 Institute of Bioinformatics and Systems Biology, National Chiao Tung University, Hsinchu, 300, Taiwan; 2 Department of Biological Science and Technology, National Chiao Tung University, Hsinchu, 300, Taiwan

## Abstract

**Background:**

The protein-protein interaction (PPI) is one of the most important features to understand biological processes. For a PPI, the physical domain-domain interaction (DDI) plays the key role for biology functions. In the post-genomic era, to rapidly identify homologous PPIs for analyzing the contact residue pairs of their interfaces within DDIs on a genomic scale is essential to determine PPI networks and the PPI interface evolution across multiple species.

**Results:**

In this study, we proposed "pair Position Specific Scoring Matrix (*pair*PSSM)" to identify homologous PPIs. The *pair*PSSM can successfully distinguish the true protein complexes from unreasonable protein pairs with about 90% accuracy. For the test set including 1,122 representative heterodimers and 2,708,746 non-interacting protein pairs, the mean average precision and mean false positive rate of *pair*PSSM were 0.42 and 0.31, respectively. Moreover, we applied *pair*PSSM to identify ~450,000 homologous PPIs with their interacting domains and residues in seven common organisms (e.g. *Homo sapiens*, *Mus musculus*, *Saccharomyces cerevisiae *and *Escherichia coli*).

**Conclusions:**

Our *pair*PSSM is able to provide statistical significance of residue pairs using evolutionary profiles and a scoring system for inferring homologous PPIs. According to our best knowledge, the *pair*PSSM is the first method for searching homologous PPIs across multiple species using pair position specific scoring matrix and a 3D dimer as the template to map interacting domain pairs of these PPIs. We believe that *pair*PSSM is able to provide valuable insights for the PPI evolution and networks across multiple species.

## Background

Many biological processes involve different types of interactions among proteins. Listing the proteins in the cell is not enough to fully understand the cellular machinery and all the interactions between them need to be delineated as well [1]. Recently, systematic identification of protein-protein interactions (PPIs) had been proposed by high throughput experimental methods (e.g. large scale yeast two-hybrid analysis and tandem affinity purification) and computational methods (e.g. phylogenetic profiles [2], gene expression profiles [3], known three-dimensional (3D) complexes [4], and interologs mapping [5]). The PPIs for diverse organisms obtained from these methods were being collected by many databases, such as DIP [6], BIND [7], MIPS [8] and STRING [9]. As an increasing number of reliable PPIs become available, a new concept and method to identify homologous PPIs across multiple species has been proposed to understand a newly determined PPI in the post-genomic era [10].

In the interactions between proteins, the protein domains interact physically with one another to perform the necessary functions. The domain-domain interaction (DDI) can recruit the formation of multi-protein signalling complexes, and control the conformation, activity, and substrate specificity of enzymes [11]. However, almost all large scale methods to explore interacting proteins cannot respond how a protein interacts with another one in molecular detail (which domains bind directly), whether experimental or computational methods. In addition, identification of interacting domain through 3D structural information provides interacting domains and atomic details for thousands of direct physical interactions between proteins [12,13]. For example, the membrane fusion adaptor p47 forms a tight complex with p97 and mediates p97 binding to its t-SNARE (soluble NSF attachment protein receptor) syntaxin5 for another round of membrane fusion [14] (Figure S1 in Additional file 1). According to the result of PSI-BLAST, both the two homologous proteins in *Saccharomyces cerevisiae *(baker's yeast) are very similar (*E*-value < 10^-100 ^and sequence identity > 30%) to the template in *Mus musculus *and might interact with each other based on the definition of homologous PPIs [10]. However, the Rix7p lacks the important binding domain (i.e. CDC48_N domain) and not involved in the process of membrane fusion although both p97 and Rix7p belongs to type II AAA+ proteins which containing two ATPase domains. Therefore, the protein pair of Rix7p and Shp1p should be not a homologous PPI of p47 and p97 due to lack of the interacting domains.

The statistical interfacial pair potentials have been used to score how well the query protein pair fit the template structure by previous methods [12,13]. This is a general empirical matrix for all the dimers of known structures to model the pair of query proteins [13]. However, although binding sites are mainly hydrophobic, protruding, and electrostatic complementary, no general patterns are observed [15]. It had been found that the free energy of binding is not evenly distributed across interfaces; instead, there are hot spots of binding energy made up of a small subset of residues in the interface of complexes [16]. There is a correspondence between the experimental identified energy hot spots and the structurally conserved residues [15]. Recently, many studies had been proven that conservative residues may perform specific functional (e.g. catalysis, recognition, binding) role [17,18]. Therefore, we consider the general empirical matrix cannot characterize all binding site correctly.

To address these issues, we proposed a new method "pair Position Specific Scoring Matrix (*pair*PSSM)" to estimate the probabilities with which residue pairs occur at various contact positions by evolutionary profiles, leading to a more sensitive scoring system. According to our knowledge, *pair*PSSM is the first method for searching homologous PPIs across multiple species using a 3D dimer as a template and automatically mapping interacting domains for these PPIs. The experimental results demonstrate that *pair*PSSM can successfully identify the homologous PPIs with 90% accuracy. Moreover, *pair*PSSM could be applied to search the homologous PPI across seven organisms commonly used in molecular research, including *Homo sapiens*, *M. musculus*, *Rattus norvegicus*, *Caenorhabditis elegans*, *Drosophila melanogaster*, *S. cerevisiae *and *Escherichia coli*. In these seven organisms, our method infers ~450,000 homologous PPIs in which the interacting domains and residues (binding sites) are automatically modelled. Based on these homologous PPIs, we believe that *pair*PSSM is able to provide valuable insights for PPI evolution and networks across multiple species.

## Materials and methods

### Pair position specific scoring matrix (*pair*PSSM)

**Figure 1:**
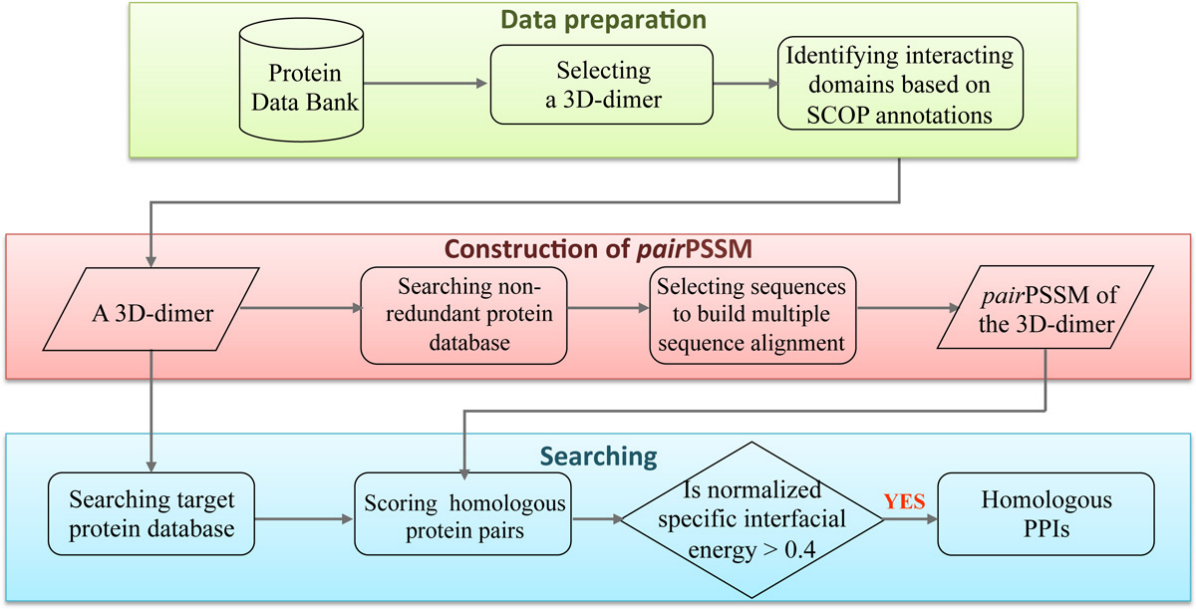
**Overview of *pair*PSSM for searching homologous protein-protein interactions**. For each 3D-dimer with SCOP annotation derived from Protein Data Bank, the *pair*PSSM is constructed to assess the fit of any possible interacting protein pairs. We use these dimers as queries to search target protein database and identify homologous protein-protein interactions of the query using the *pair*PSSM.

Figure 1 shows the overview of the *pair*PSSM to search homologous PPIs using a 3D-dimer as the template. First, we collected non-redundant 3D-dimers from Protein Data Bank (PDB) and identified interacting domains based on the SCOP database. For each 3D-dimer, we estimated the probabilities with which residue pairs occur at various contact positions and constructed a *pair*PSSM for assessing the fit of any possible interacting protein pairs. Next, we used the dimer as a query to search candidates of homologous PPIs from the target protein sequence database (e.g. *S. cerevisiae*). Finally, the *pair*PSSM is used to calculate the normalized specific interfacial energy of each candidate for identifying homologous PPIs of the query. The energy of each homologous PPI calculated from the *pair*PSSM and empirical matrix are the specific interfacial energy and general interfacial energy, respectively. The details of *pair*PSSM construction for each 3D-dimer are descripted by the following steps.

#### Scoring matrix architecture

For a 3D-dimer with the number of contact residue pairs *R*, the empirical matrix of dimension 20 × 20 is replaced by a protein-protein interaction position-specific matrix (*pair*PSSM) of dimension *R *× 20. A typical *pair*PSSM with 47 (*R *= 47) contact residue pairs was shown in Figure S2 in Additional file 1. The residue pair in a contact position is considered as a single symbol. The advantage of this matrix is estimation of the probabilities with which residue pairs occur at various contact positions, leading to a more sensitive scoring system.

#### Construction of evolutionary profile

To obtain the evolutionary profiles from multiple sequence alignment, our alignment result should be come from a comprehensive and non-redundant protein database. The protein database is obtained from the NCBI Reference Sequences database (RefSeq). Although RefSeq aims to provide a non-redundant set of sequences for users, two major sources [i.e. alternative splicing and duplication of genes (paralog)] of redundancy occur in RefSeq. Therefore, we used BlastCluster to remove redundancy with 90% identical over an area covering 90% of the length of each sequence in the same species. Finally, 2,109,945 protein sequences were selected into our non-redundant protein database.

To produce a multiple sequence alignment from the PSI-BLAST, we collected all RefSeq sequence segments aligned to the two proteins of 3D-dimer with *E*-value ≤ 10^-9^. The two proteins of 3D-dimer are used as a template for constructing two multiple sequence alignments, respectively. Any sequence that is > 95% identical to 3D-dimer (template) is purged.

#### Target frequency estimation

Given two multiple sequence alignment derived from a 3D-dimer, we generated score matrices with the theoretical foundation for the score of a specific contact position using the form log (*Q_ij_/P_ij_*), where *Q_ij _*is the estimated probability for the contact residue pair *i&j *to be found in the column; *P_ij _*is the expect probability of *i&j *to be found in the column. The estimate of *Q_ij _*for a specific contact position should converse simply to the observed frequency of the residue pair *i&j *in that column. To estimate the *Q_ij_*, we implemented the data-dependent the pseudocount method [19] which is relative simplicity and often performs nearly as well comparing the Dirichlet mixtures [20]. The *Q_ij _*is given as $Q_{ij}=\frac{\alpha f_{ij}+\beta g_{ij}}{\alpha +\beta }$, where *f_ij _*is observed frequency and $g_{ij}=P_{i}P_{j}e^{S_{ij}}$ is the residue pair pseudocount frequency. The *S_ij _*is the interacting energy of the residues *i&j *in empirical matrix. *P_i _*and *P_j _*are the background probabilities of residues *i *and *j*, respectively. The α and β are the relative weights given to observed and pseudocount residue frequency. In our study, α is set to the number of different residue-pair types in column -1 and β = 5. The *P_ij _*is defined as *P_ij _*= *P_i _*× *P_j_*. The residue composition of the protein interface is obtained from Lu *et al. *[21] (Table S1 in Additional file 1).

#### Amino acid classification

**Figure 2:**
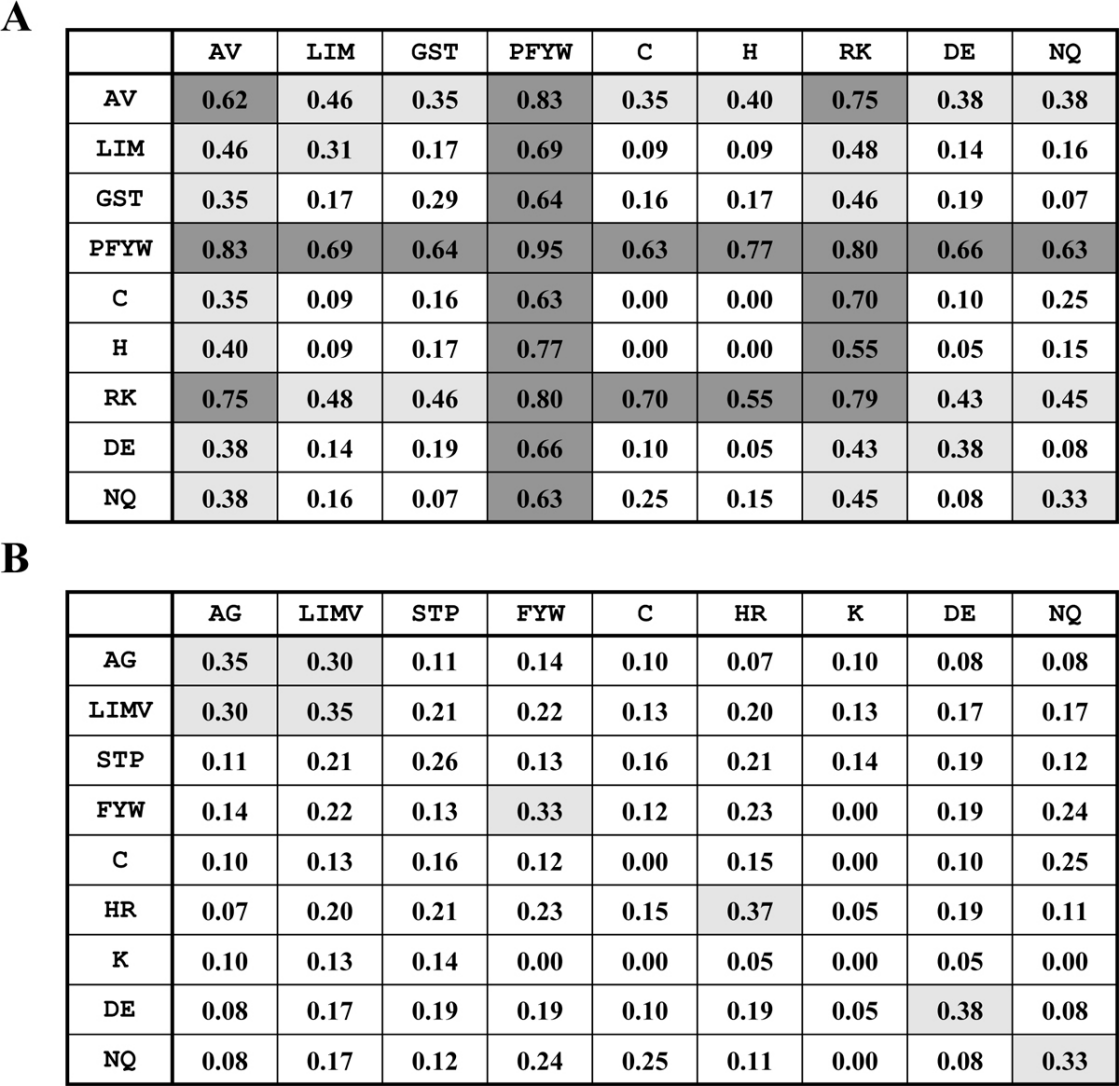
**The standard deviations of contact residue potentials in the clusters of amino acid**. The deviation > 0.5 is colored by dark gray and the deviation between 0.3 and 0.5 is colored by gray. (A) The amino acid classification is defined by Saha *et al. *(B) The classification is slightly modified by us.

The sequence variability at each contact position can be estimated based on two multiple sequence alignments of a dimer template. Unlike unconservative mutations, conservative ones preserve the essential nature of the side chain. Therefore, we make some tolerances for such mutations. Saha *et al. *[22] made a classification based on the similarity of the environment of each amino acid residue in protein structures to the nine groups: (*i*) Ala and Val; (*ii*) Met, Leu and Ile; (*iii*) Gly, Ser and Thr; (*iv*) Pro, Phe, Tyr and Trp; (*v*) Cys; (*vi*) His; (*vii*) Arg and Lys; (*viii*) Asp and Glu; (*ix*) Asn and Gln. We examine this classification of amino acid whether suitable for access the contact residue potential by calculating the standard deviation of contact residue potential in the cluster of amino acid. Figure 2A shows that the three groups [A, V], [P, F, Y, W] and [R, K] have high standard deviations of intra contact residue potential. Therefore, we slightly modify the group as follows: (*i*) Ala and Gly; (*ii*) Val, Met, Leu and Ile; (*iii*) Pro, Ser and Thr; (*iv*) Phe, Tyr and Trp; (*v*) Cys; (*vi*) His and Arg; (*vii*) Lys; (*viii*) Asp and Glu; (*ix*) Asn and Gln. In this way, all the standard deviations of intra-contact residue potential are smaller than 0.4 (Figure 2B). We considered this amino acid classification is more reasonable for measuring the contact residue potential.

### 3D-dimers and related 3D-dimers

Here, we select the co-crystallized proteins from the PDB [23] with using the following criteria. (1) The resolution of the co-crystallized protein should be smaller than 3.0Å; (2) each chain of the co-crystallized proteins should be comprised more than 35 amino acids. If the protein consists of the cross-chain domain defined by SCOP, we regard it; (3) the number of interacting residue pairs is set to be greater than 25 and each chain must contain more than 5 contact residues to make sure that the dimer is reasonably extensive [24]. Interacting residue pairs are defined as a pair of residues from different chains that have at least one pair of heavy atoms within 4.5Å with each other; (4) elimination of artificial packing complexes rather than biologically functional multimers by using PQS server [25]; (5) we remove redundancy by the sequence identity > 50%. Finally, the non-redundant template set, termed NRT, consists of 1,122 heterodimers and 3,514 homodimers.

To model PPIs, we explored whether the two similar dimers possess the similar protein interaction type. Here, we defined that two 3D-dimers contain the same interacting SCOP domain (more than three contact residues within the domain boundary) are "related-dimer pairs". Among 29,369 dimers derived from PDB, we first remove the dimers without annotations of SCOP domain then selected 5,553 heterodimers and 15,026 homodimers. Second, the dimers are clustered by BlastCluster [26] according 80% identical over an area covering 80% of the length of each sequence. We choose one representative dimer from each cluster if the number of interacting residue pairs more than the mean of the cluster and the resolution of crystallization is smallest. Third, the representative dimers are grouped based on the domain definition in SCOP. We group the dimers which possess the same interacting domain pair in family level. Totally, there are 189 groups of heterodimer and 489 groups of homodimer with ≥ 1 member. We choose one representative member for each group and pair the representative one for the all other members in the group. These pairs of dimers are considered as related dimer pairs. In this way, we derived a related 3D-dimer set, termed RD, with 459 and 1,412 related dimer pairs from the 189 groups of heterodimer and 489 groups of homodimer, respectively.

### True protein complexes and unreasonable protein pairs

Our method utilized a 3D-dimer to model all candidates of homologous PPIs and used a specific *pair*PSSM to determine whether two proteins interact. Here, we constructed two data sets, including the protein pairs form complexes (i.e. PPIs) and the protein pairs cannot interact with each other (the unreasonable protein pairs). From 189 representative 3D-dimers (excluding the antibody-antigen complex), we used the PSI-BLAST to remove the similar dimers with > 80% sequence identity and get protein pair with *E*-value ≤ 0.1. If the protein pair is co-crystallized in PDB and it contains the same interacting SCOP domains for the query dimer, we consider the protein pair as the positive (i.e. true protein complex) as the homologous PPI of the 3D-dimer. In the other word, if the protein pair is not co-crystallized in PDB and it does not contain the same interacting SCOP domain for the query dimer, we considered the protein pair as negative cases (i.e. unreasonable protein pairs). Among 189 representative 3D-dimers, we selected 224 pairs of positive homologous PPIs and 282 pairs of negative protein pairs.

### Gold standard positives and negatives of PPIs in *S. cerevisiae*

To examine the *pair*PSSM for identifying homologous PPIs in *S. cerevisiae*, the *S. cerevisiae *proteome was obtained from the *Saccharomyces Genome Database *(SGD) [27]. The corresponding amino acid sequences of total 5,877 open reading frames (ORFs) were collected. 5,882 reliable PPs, considered as gold positive set, were downloaded from the core set of *Database of Interacting Protein *(DIP) [6]. For gold negative set, we followed the previous study [3] assumed that the proteins in different compartments do not interact with each other and generated 2,708,746 non-interacting protein pairs according to the proteins in separate subcellular compartments [28].

### Yeast gene expression

The gene expression profiles of two interacting proteins were also used to access the accuracy of our method according to the basic assumption: "the gene pair with similar expression profiles is likely to encode an interacting protein pair" [29]. The Rosetta compendium set consisting of the expression profiles of 300 deletion mutants and under chemical treatments [30] was used to measure the similarity of gene expression profiles of two genes.

### Performance criteria

To assess the quality of our method, the mean average precision (MAP) and mean false positive rate (MFP) are used in this study. The MAP is defined as $MAP=\left(\sum_{j=1}^{M}AP_{j}\right)/M$, where $AP=\left(\sum_{i=1}^{A}i/T_{i}\right)/A;T_{i}$ is the number of protein pair candidates in a hit list containing *i *positive PPIs; ***A ***is total numbers of positive PPIs in the template *T*; ***M ***is the total numbers of template. The MFP is defined as $MFP=\left(\sum_{j=1}^{M}FP_{j}\right)/M$ where $FP=\left(\sum_{i=1}^{A}\left(T_{i}-i\right)/\left(K-A\right)\right)/A;K$ is the total numbers of protein pair candidates in template *T *from PSI-BLAST.

In addition, the similarity of two gene-expression data is defined by the Pearson correlation coefficient between the two gene-expression profiles. To test whether the mean of correlation coefficient for candidates of protein-protein interactions higher than that of non-interacting protein pairs, we calculate the T-score and the P-value for the null hypothesis of the sample mean (our prediction) smaller than the mean of gold negative set.

## Results and discussion

We first explore the relationship between sequence similarity and interacting similarity for homologous PPIs. Modelling PPIs by homology is reasonable only when the correlation is high enough. Second, the *pair*PSSM is verified in two data sets: (1) we examined whether the energy calculated from *pair*PSSM could distinguish the true protein complexes and unreasonable protein pairs; (2) for identified homologous PPIs in yeast proteome through *pair*PSSM, we used two common metrics (i.e. MAP and MFP) to assess the performance and compared with the empirical matrix used by previous method. Third, the similarity of gene expression profiles for the candidates of protein-protein interactions is examined. Finally, we applied *pair*PSSM to identify homologous PPIs for seven organisms and used two biological examples to illustrate the operation and power of *pair*PSSM.

### Similar 3D-dimers imply similar interacting types

The RD set (459 pairs of related heterodimers and 1412 pairs of related homodimers) described above provides all instances of a particular interaction type occurring within different complex structures, that we then wish to compare to each other and correlate with sequence similarity. To compare the binding of different instances of the two dimers with the same interacting domains, we used pair coverage (*PC*) to calculate binding site overlap from the number of shared interacting residue pairs. Given a pair of related dimers A-B and A'-B', where A-A' and B-B' contain same SCOP domains. we use a structural alignment tool, CE [31], to align the A-A' and B-B', respectively. The *PC *is defined as $PC=\sqrt{\frac{NCPM^{2}}{NCP_{AB}\times NCP_{A^{\prime }B^{\prime }}}}$, where the *NCPM *is the matching number of contact residue pairs between the structural alignment of A-A' and B-B'; *NCP_AB _*and NCP*_A'B' _*are the number contact residues pairs of dimer A-B and A'-B', respectively.

The interacting types of two dimers are very alike when the *PC *of a related dimer pair is greater than 0.4. In following discussion, the sequence identity between two dimers is defined as the minimum sequence identity in A&A' and B&B'. The rationale is that the interacting partners with the lower sequence identity would tend to be the better indicator for the diversity of the interaction.

**Figure 3:**
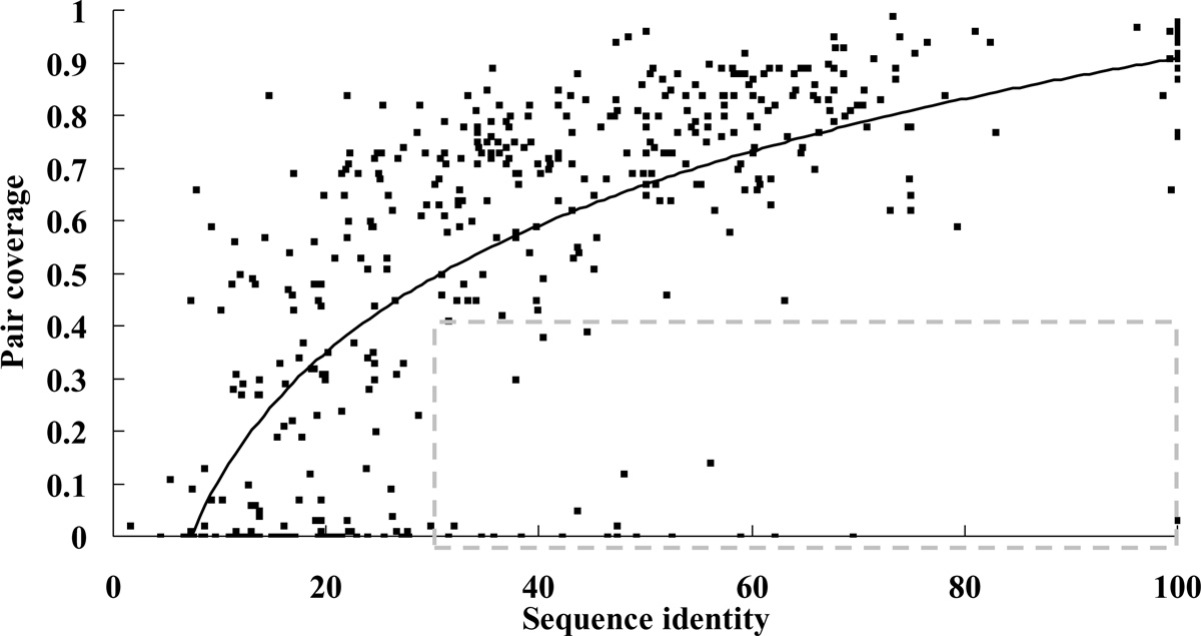
**The relationship between sequence identity and pair coverage (*PC*) of 459 pairs of related hetero dimers**. Among 459 pairs of related heterodimers, the sequence identities of 280 related heterodimer pairs are > 30%. In 280 related heterodimer pairs with > 30% sequence identity, there are 91% (256/280) pairs with *PC *≥ 0.4. The rate of exception (*PC *< 0.4) is only 9%. The dots in gray box are the exceptions of the pairs with > 30% sequence identity but pair coverage < 0.4.

Figure 3 shows the distribution of pair coverage with different minimum sequence identity for 459 pairs of related hetero dimers. It is clear that the interactions tend to be similar when sequence identity is above 30%. Among 280 pairs of related heterodimers with > 30% sequence identity, there are 91% (256/280) pairs with *PC *≥ 0.4. The rate of exception (*PC *< 0.4) is only 9% (24/280) (Table S2 in Additional file 1). Surprisingly, 11 out of 24 cases contain the b.1.1.1 domain (V set antibody variable domain). This result means that the interacting types of antigen-antibody complex are not conserved. We proposed an example of two much similar antigen-antibody complex but their interacting types are completely different (Figure S3 in Additional file 1).

In the pairs of related homodimers, the trend which gives a guide to the degree of sequence similarity needed to be confident in a similar interaction is also observed. However, there are more exceptions (~30%) in the pairs of homodimers than heterodimers (Figure S4 in Additional file 1). In addition, the *PC*s of heterodimers are higher than that of homodimers in difference sequence identity (Figure S5 in Additional file 1). The result means that the specificity of interaction in heterodimer is more conserved than homodimers. For this reason, we think that heterodimers are more suitable for the template to model PPIs than homodimers.

In summary, we find the related dimers indeed keep similar interacting type. Sequence similarity needed to be confident in a similar PPI. We suggest one must be careful with identity below than 30% to model PPIs by homology. Because the specificity of PPI in heterodimer is more conserved than homodimers, we consider the heterodimers are more suitable for the templates to model interactions. Finally, we find that the interacting types of antigen-antibody complexes are often diverse, they may be not suitable for used to as templates.

### Verification in true protein complexes and unreasonable protein pairs

To verify *pair*PSSM, we would likely to study whether the specific interfacial energy calculated from *pair*PSSM could distinguish the true protein complexes (positive set) and unreasonable protein pairs (negative set). In 224 pairs of positive homologous PPI (i.e. true protein complexes) and 282 pairs of negative protein pair (i.e. unreasonable protein pairs), we used one dimer to score the other one by our specific empirical matrix (*pair*PSSM) and general empirical matrix. Based on the frequency of positive set and negative set occurred in different specific interfacial energy intervals, we found that the interfacial energy of positive set is significant higher than that of negative set (Figure S6A in Additional file 1). Next, the error rate is calculated by averaging the number of false positive divided by number of positive set and the number of false negative divided by number of negative set. Experimental result shows that the minimum error rate is 18% when the specific interfacial energy (*pair*PSSM) is set to 50 (Figure S6B in Additional file 1).

In addition, we also applied the general interfacial energy based on empirical matrix to the positive set and negative set (Figure S7 in Additional file 1). When the general interfacial energy is set to 10, we can obtain the minimum error rate 17%. Interestingly, the distributions of positive and negative dataset are not high concentrated in the two sides (positives in high energy and negatives in low energy) when using the specific interfacial energy (Figure S6A in Additional file 1). That is why the error rate higher in using specific interfacial energy than in using general interfacial energy. In addition, the correlation (0.9321) between the specific interfacial energy and the number of contact residues (Figure S8 in Additional file 1) is higher than the correlation (0.6753) between general interfacial energy and the number of contact residues (Figure S9 in Additional file 1).

Because specific interfacial energy is highly dependent on the characteristic of dimer template, we design a method to normalize the specific interfacial energy. When a protein pair is modelled by a 3D-dimer and gets a specific interfacial energy scored by *pair*PSSM, we normalize the energy defined as the specific interfacial energy of the protein pair divided by the specific interfacial energy of the dimer template. By using the normalized interfacial energy, we can find the distribution of positive and negative dataset is much more concentrated in the two sides (Figure S10A in Additional file 1) than the unnormalized (Figure S6A in Additional file 1) and the error rate reduce from 18% to 13% (Figure S10B in Additional file 1). For this reason, we consider the normalized specific interfacial energy equal set to 0.4 is a good threshold for identifying homologous PPIs.

### Verification in yeast proteome

Here, we identified homologous PPIs in *S. cerevisiae *and used the average precisions and false positive rate, commonly used to evaluate the quality of database searching, to verify our *pair*PSSM. In the NRT set, 1,122 representative heterodimers were considered as queries to search database of yeast proteome by PSI-BLAST. We defined the proteins searched out with *E*-value ≤ 10^-3 ^are homologous to the query protein. Given a query of heterodimer A-B, A' and B' are the homologous proteins of A and B, respectively. All the homologous protein pairs A'-B' are considered as candidates of homologous PPIs. Among these candidates, the known interacting protein pairs and the others are considered as positives and negatives, respectively.

**Figure 4:**
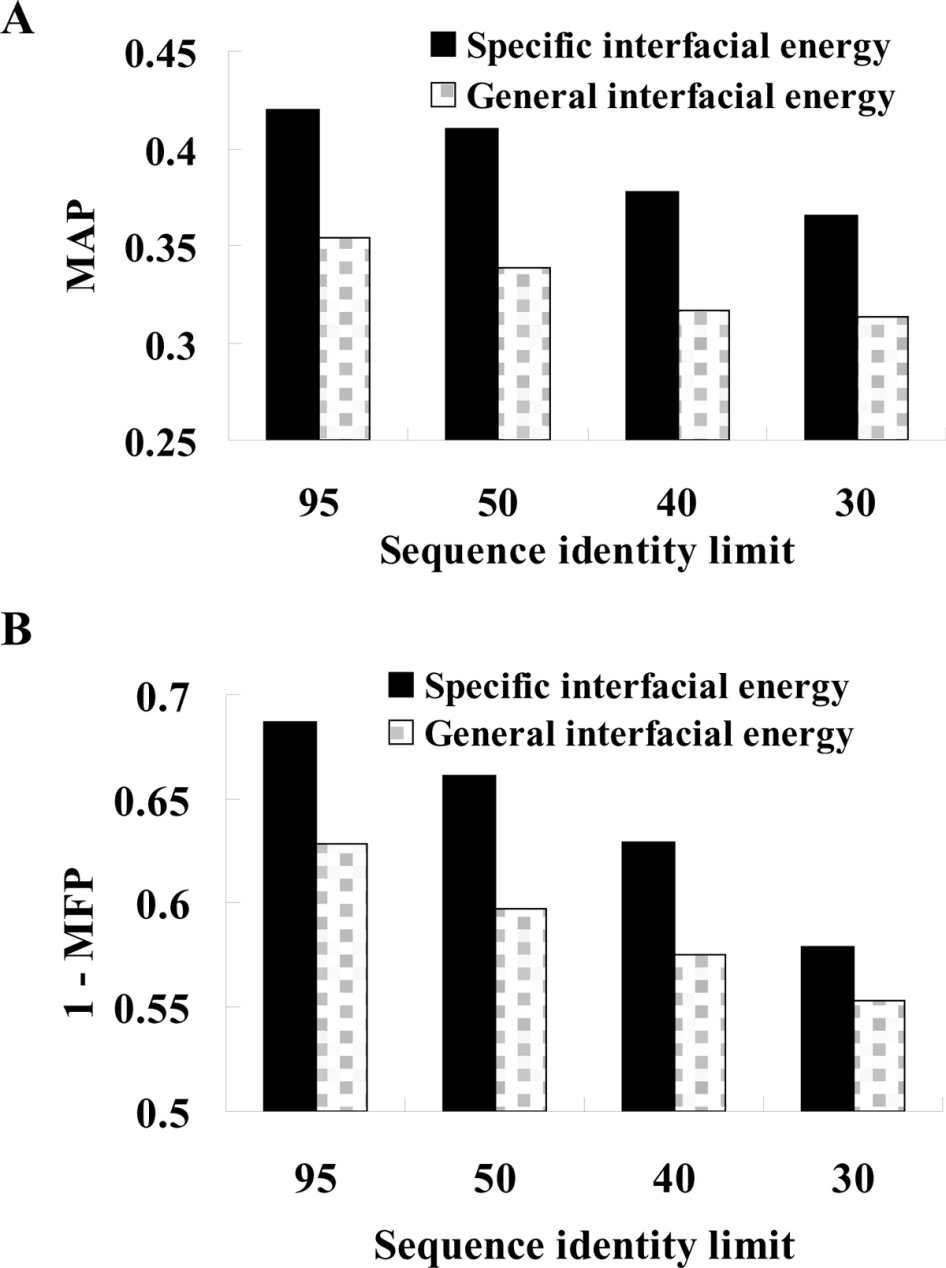
**The mean average positions and mean false positive rate of 182 queries**. The (A) MAP and (B) 1-MFP distributions between specific interfacial energy and general interfacial energy in sequence identity limit with 95%, 50%, 40% and 30%. The unannotated candidates are considered as negatives. Sequence identity limit means that if one protein of candidate with sequence identity > sequence identity limit, the candidate is removed.

Among 1122 queries, 182 queries have both positive candidates and negative candidates, and then these queries could calculate the average precisions and false positive rate (Table S3 in Additional file 1). Figure 4 shows the mean average precisions (MAP) and mean false positive rate (MFP) of the 182 queries with different sequence identity limit. The MAP and MFP are 0.42 and 0.31 by using specific interfacial energy, respectively. On the other hand, the MAP is 0.35 and the MFP is 0.37 by using the general interfacial energy. In order to avoid our method merely identify homologous PPIs with high sequence identity, we set the sequence identity limit to remove the candidates if one protein of candidates with sequence identity > sequence identity limit. These experimental results indicate the *pair*PSSM is much better than the general empirical matrix even though in identifying remote homologous PPIs (i.e. lower sequence identity).

In the above results, the candidates which are not included in the known interacting protein pairs are considered as negatives. However, it may be somewhat unreasonable because many candidates are indeed interacting proteins in nature but have not proven by experimental methods in the past. Here, we considered the candidates overlapping with 2,708,746 non-interacting protein pairs defined by Jasen *et al. *as negatives [28]. The candidates without any annotations are removed for calculate average precisions and false positive rates. In this way, our method using *pair*PSSM is about 10% improvement than using general empirical matrix (Figure S11 in Additional file 1). In the future, we will compare our pairPSSM with previous works [4,12,13] carefully to judge the utilities and advantages for predicting PPIs.

### Example analysis: PDB code 1a2kAD

**Figure 5:**
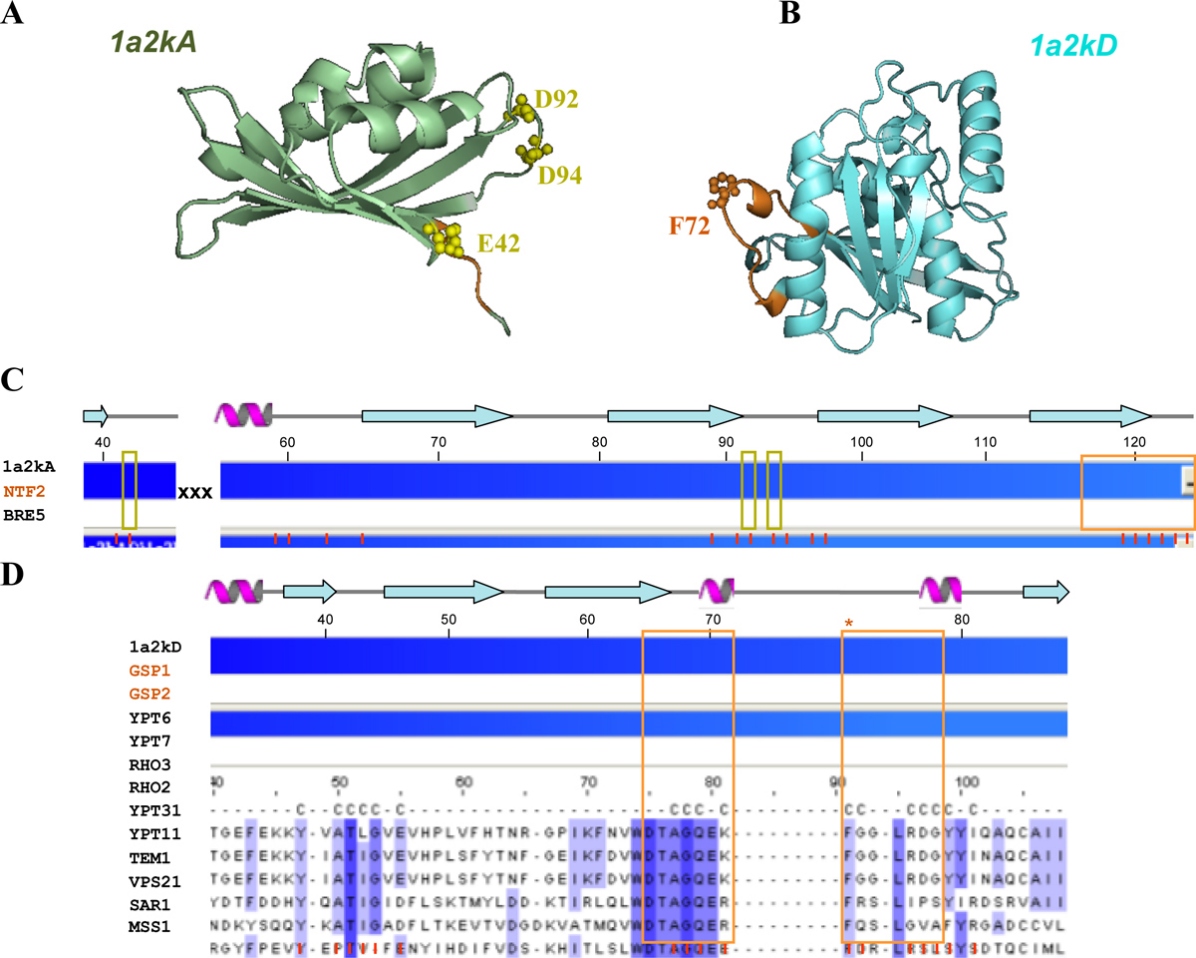
**The 3D-structure of 1a2kAD and multiple sequence alignment results of the 14 candidates to their corresponding template proteins of 1a2kAD**. (A) A chain of 1a2k. The three important negatively charged residues, Glu42, Asp92 and Asp94, are colored by yellow. The C terminal peptide is colored by orange. (B) D chain of 1a2k. The switch II loops is colored by orange. (C) Alignment result of 1a2k A chain. Three important negative residues are marked in the yellow box. The hydrophobic peptide in C terminal is also an important interactive site (orange box). The red bars in the bottom are the contact positions in 1a2k A chain. (D) Alignment result of 1a2k D chain. The switch II loops are marked in orange box. The important aromatic residue Phe72 is marked by orange star. The red bars in the bottom are the contact positions in 1a2k D chain.

We give an example using the 3D-dimer, PDB code 1a2kAD, to search database of yeast proteome and illustrate the accuracy and operation of *pair*PSSM. The A chain of 1a2k is a rat nuclear transport factor 2 (NTF2) and the D chain of 1a2k is a dog GTP binding protein ran (Ran) [32]. The transportation between nucleus requires to the nuclear pore complexes (NPC) in the nuclear envelope and several key factors including importin α and β, which recognize proteins with a nuclear localization sequences (NLS), the small GTP binding protein ran and nuclear transport factor (NTF) [33,34]. For this 3D-dimer (1a2kAD) as query (Figures 5A and 5B), we obtain 14 protein pair candidates (Table S4 in Additional file 1). The two protein pair candidates, NTF2&GSP1 and NTF2&GSP2, were proven to bind with each other by yeast two hybrid test [35,36] and the other twelve protein pair candidates are non-interacting proteins due to locate in different compartments. Interestingly, the UBP3-associated protein BRE5 (BRE5), one of the proteins in these negative protein pairs, and the NTF2 have the same domain annotation (PF02136, NTF2 domain) [37]. Based on two-hybrid assay, the NTF2 domain of BRE5 is necessary and sufficient to interact with ubiquitin carboxyl-terminal hydrolase 3 [38]. In addition, NTF2 and BRE5 perform nucleocytoplasmic transport and coregulate vesicle transport in cytoplasm and nuclear envelope, respectively. These results show that BRE5 with transport functions may be the paralogous protein of NTF2 but does not interact with the proteins (e.g., GSP1 and GSP2), because BRE5 and the proteins of these negative protein pairs are in different subcellular compartments (Table S4 in Additional file 1). The normalized specific interfacial energies calculated from *pair*PSSM (Figure S2 in Additional file 1) of the two positive protein pairs are both above the threshold 0.4 and the twelve negative protein pairs are below the threshold (Table S4 in Additional file 1). The result shows that *pair*PSSM is useful for identifying homologous PPIs. However, the general interfacial energies of the two positive protein pairs and ten out of twelve negative protein pairs are both above the threshold -15 (the more negative are more favor to bind) [21]. In summary, 12 out of 14 protein pairs are identified incorrectly with general interfacial energy and all the 14 PPIs are identified correctly by our specific interfacial energy.

The multiple sequence alignment results between the 14 candidates and their corresponding template, such as A chain of 1a2k (Figure 5C) and D chain of 1a2k (Figure 5D). The interface involves primarily the putative switch II loop of ran (residue 65 to 78) (Figure 5B, orange box) and the hydrophobic cavity surrounding surface of NTF2 [32]. The interaction made by the switch II loops accounts for the ability of NTF2 to discriminate between GDP and GTP bounds forms of Ran. A striking feature of the interacting interface was the aromatic ring of Phe72 of Ran (Figure 5B, orange residue and Figure 5D, orange star site). It inserts into the hydrophobic cavity of NTF2 where it was surrounded by the hydrophobic side chains of Trp41, Leu59, Phe61, Ile64, Leu89, Ala91, Met97, Phe119 and Leu121. The positive protein pairs (i.e. NTF2&GSP1 and NTF2&GSP2) are both conservative in this important site (Figure 5). On the other hand, the interacting interface on NTF2 involved this molecule's characteristic hydrophobic cavity. Hydrophobic residues in the upper portion of the NTF2 cavity, together with negatively charged residues, Glu42, Asp92 and Asp94, are surrounding the cavity (Figure 5A, yellow residues and Figure 5C, yellow boxes) made significant contributions to the interface with GDP-Ran. The three important negatives residues are conservative from A chain of 1a2k (rat NTF2) to the yeast NTF2. However, the three important sites are mutated to Threonine in BRE5 (Figure 5A). The BRE5 is an ubiquitin protease cofactor which forms deubiquitination complex with ubp3p that coregulates anterograde and retrograde transport between the endoplasmic reticulum and Golgi compartments. The three important residues mutated may be resulted in BRE5 does not interact with GSP1 and GSP2. Encouragingly, we give poor score to these two candidates (0.08) and successfully identify the true homologous PPIs (i.e. GSP1&NTF2 and GSP2&NTF2).

### Verification in yeast expression profiles

Recently, many scientists consider that genes with similar expression profiles are likely to encode interacting proteins [39]. Therefore, we compare the distribution of gene expression profiles for the two gold standard sets and our identified homologous PPIs by *pair*PSSM with normalized specific interfacial energies ≥ 0.4 and 0.6 (Figure S12 in Additional file 1). The homologous PPIs composed of the same protein are not used to calculate the gene expression profiles because their expression profiles must be identical and should not be taken account of. Experimental result shows that the distribution of the correlation coefficients of our identified homologous PPIs is similar to the core set of DIP (positives) and right shift to non-interacting protein pairs (negatives) (Figure S12 in Additional file 1). Then, we used standard two sample T-test to test the mean of correlation coefficient for our identified homologous PPIs to non-interacting protein pairs. The *E*-values of the two sets are 10^-30 ^and 10^-26^, respectively. These results indicate that the mean of correlation coefficient for identified homologous PPIs by *pair*PSSM is significant higher than that for non-interacting protein pairs.

### Identification of homologous protein-protein interactions in seven common organisms

In the above section, we have verified our *pair*PSSM in two data sets and obtained a reasonable threshold for normalized specific interfacial energy about 0.4~0.5. Here, we apply *pair*PSSM to identify homologous PPIs in seven organisms commonly used in molecular research, including *H. sapiens*, *M. musculus*, *R. norvegicus*, *C. elegans*, *D. melanogaster*, *S. cerevisiae *and *E. coli*. By set the threshold to 0.5, we obtain about 450,000 homologous PPIs from the seven common organisms (Table S5 in Additional file 1). Comparing our identified homologous PPIs and the PPIs deposited in DIP database, there is a large difference for number of interactions in the same organism. For example, we identify 1,850 PPIs in *S. cerevisiae *but DIP collects 25,165 PPIs. On the other hand, we identify 223,151 PPIs in *Homo sapiens *but DIP collects only 12,975 PPIs. There are two reason for large drop, one is the large-scale experimental method (such as yeast two-hybrid analysis or proteomics-immunoprecipitation) is hard to apply in mammalian organisms and results in the interactions deposited in DIP are few in *H. sapiens*, *M. musculus*, or *R. norvegicus*. The other reason is gene duplication and alternative splicing often occurred in the mammalian organisms and result in some redundancy protein in protein database. In these organisms, we may over estimate the number of identified PPIs.

Structural genomics projects are generating new structures at an unprecedented rate--a benefit of recent developments in high-throughput technologies [40]. As a result, the number of protein structures in the Protein Data Bank (PDB) is increasing rapidly. For each new determined 3D-dimer, we can apply our method to identify all the candidates in thousands of organisms quickly. It helps the biologists to further detail analysis the PPI network.

### Model homologous protein-protein interactions in *H. sapiens *by 1evtBD

The *pair*PSSM can apply not only to yeast proteome but also to any other organisms. PPIs between the fibroblast growth factors (FGFs) and their receptors had been intensive studied [41,42]. FGFs play key roles in morphogenesis, development, angiogenesis, and wound healing. These FGF-simulated processes are mediated by for FGF receptor tyrosine kinase. There are more than 20 human protein FGFs that bind to one or more of 7 FGF receptors (FGFR1b, -1c, -2b, -2c, -3b, -3c, -4), where the c and b denote isoforms IIIc & IIIb formed by alternative splicing. The complex of FGF1/FGFR1 (Figure S13 in Additional file 1) had been dissolved by Plotnikov *et al. *(PDBID: 1evt) [43]. Ornitz *et al. *perform a study of FGFR specificity by measuring mitogenic activity of FGFR-inducible BaF3 cell-line [44]. Based on the binding affinity of the seven FGF/receptor complexes (from FGF4 to 7 receptors, FGFR1b, -1c, -2b, -2c, -3b, -3c, -4) [44] (Table S6 in Additional file 1), we determined that low and high binding affinity relative to the FGF1 are < 10% and > 10%, respectively.

In this study, we used the *pair*PSSM of 1evtBD to model homologous PPIs for the seven FGF/receptor complexes. Among seven FGF/receptor complexes, six complexes are high affinity and our pairPSSM also give high interfacial energy. However, the other one, FGF4/FGFR3b complexes, with very low binding affinity (1.0%) but our pairPSSM give a high normalized interfacial energy (0.84). For the detailed sequence analysis (Figure S13 in Additional file 1), we find that most contact positions in FGFR3b are much conservative except some residues in D3 immunoglobulin (Ig)-like domains (Figure S13 in Additional file 1, orange box). This result may mean some other factors involved in determining the strength of the FGFR interactions. In conclusion, we successfully identify 6 out of 7 FGF/receptor complexes. There is a good agreement between the specific interfacial energy and binding affinity even though still with an incorrect case.

## Conclusions

We have developed a new method "*pair*PSSM", a more sensitive scoring system for estimating the probabilities with residue pairs occurred at various contact positions by evolutionary profiles, to infer domain annotated homologous PPIs across multiple species. The specific interfacial energy calculated from *pair*PSSM can successfully distinguish the true protein complexes and non-reasonable protein pair with about 90% accuracy. Experimental results show that the *pair*PSSM outperforms general empirical matrix about 10% improvements even though for the distantly related protein sequences. Moreover, we applied *pair*PSSM to identify ~450,000 homologous PPIs, automatically modelled the interacting domains and residues (binding sites), in seven organisms, including *H. sapiens*, *M. musculus*, *R. norvegicus*, *C. elegans*, *D. melanogaster*, *S. cerevisiae *and *E. coli*. Based on these homologous PPIs, we believe that *pair*PSSM is able to provide valuable insights for PPI evolution and networks across multiple species.

## Competing interests

The authors declare that they have no competing interests.

## Authors' contributions

CYL, YCC, and JMY conceived and designed the experiments. YCC, YSL, and JMY implemented the program. CYL, YSL, and JMY performed the experiments and analyzed the data. CYL, YCC, and JMY wrote the paper.

## Declarations

The publication costs for this article were funded by National Science Council, partial supports of Ministry of Education and National Health Research Institutes [NHRI-EX100-10009PI].

This article has been published as part of *BMC Bioinformatics *Volume 14 Supplement 2, 2013: Selected articles from the Eleventh Asia Pacific Bioinformatics Conference (APBC 2013): Bioinformatics. The full contents of the supplement are available online at http://www.biomedcentral.com/bmcbioinformatics/supplements/14/S2.

## Supplementary Material

Additional file 1 **The supplementary information**. Click here for file
