# Supplementary material for: Inferring homologous protein-protein interactions through pair position specific scoring matrix
Source: BMC Bioinformatics. 2013 Jan 21;14(Suppl 2):S11. doi: 10.1186/1471-2105-14-S2-S11 (PMC3549806; doi:10.1186/1471-2105-14-S2-S11)
Supplement: Additional file 1 — The supplementary information. [file 1471-2105-14-S2-S11-S1.pdf]

Additional files

A

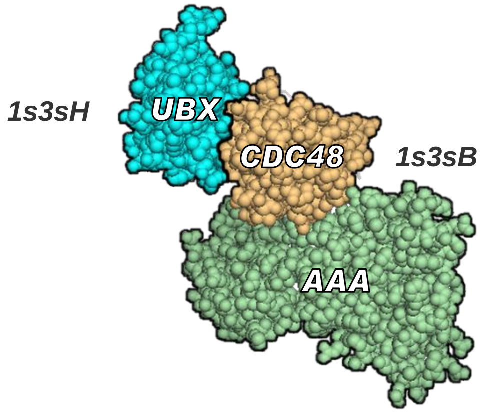

B

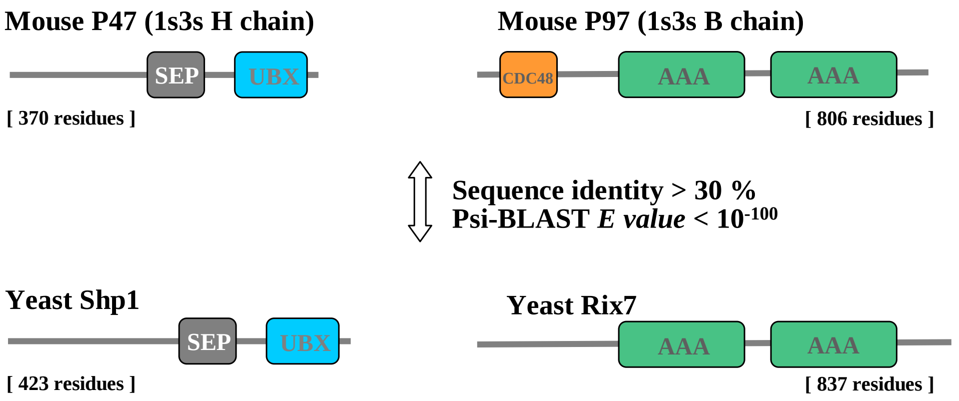

Supplementary Figure S1 - The 3D structure and domain architecture of protein complex P47/P97.

(A) The 3D structure of protein complex P47/P97. (B) The domain architecture of two mouse proteins P47&P97 and two yeast proteins Shp1&Rix1. Both protein pairs (P47 to Shp1 and P97 to Rix7) are with sequence identity > 30% and PSI-BLAST *E*-value $< 10^{-100}$. The color boxes are represented as functional domains.

Contact position		AA	AB	AC	AD	AE	AF	AG	AH	AI	BB	BC	BD	BE	BF	BG	BH	BI	CC	CD	CE	CF	CG	CH	CI	DD	DE	DF	DG	DH	DI	EE	EF	EG	EH	EI	FF	FG	FH	FI	GG	GH	GI	HH	HI	II
1	DD	-1.0	0.1	-1.0	0.5	1.0	-0.5	-1.7	-1.7	-1.1	1.7	0.0	1.9	2.0	0.5	-0.6	-0.6	0.0	-0.9	0.6	1.0	-0.5	-1.5	-1.4	-1.0	5.3	2.3	0.9	-0.1	-0.1	0.3	4.2	1.5	0.3	-0.2	0.6	-0.1	-1.3	-0.3	-0.5	-2.2	-1.1	-1.6	-2.2	-1.5	-0.9
2	HD	-1.2	-0.1	-1.2	0.3	0.8	-0.8	-1.9	-1.9	-1.4	1.5	-0.2	1.6	1.7	0.3	-0.8	-0.4	-0.3	-1.2	0.5	0.8	-0.7	-1.7	-1.4	-1.2	1.9	2.1	0.7	1.0	3.3	0.1	4.0	1.3	0.0	-0.4	0.4	-0.3	-1.5	-0.6	-0.8	-2.5	-1.3	-1.8	-2.4	-1.7	-1.2
3	HA	-1.5	-0.4	-1.1	-0.1	0.5	-1.1	-2.3	1.3	-1.7	1.1	-0.5	1.2	1.4	-0.1	-1.1	-1.2	-0.6	-1.5	0.0	0.4	-1.0	-2.1	-0.5	-1.5	1.6	1.7	0.3	-0.7	-0.7	-0.2	3.7	1.0	-0.3	-0.8	0.1	-0.7	-0.2	2.3	-1.1	-0.5	-0.8	-1.1	-1.6	-2.1	-1.5
4	HF	-1.5	-0.4	-1.6	-0.1	0.5	-1.1	-1.2	0.6	-1.7	1.1	-0.5	1.2	1.4	-0.1	-1.1	-0.8	-0.6	-1.5	0.0	0.4	-0.6	-0.9	1.3	-1.5	1.6	1.7	0.3	-0.7	-0.7	-0.2	3.7	1.0	-0.3	-0.8	0.1	-0.7	-0.7	1.8	-1.1	-2.8	-0.8	-2.1	-2.7	-2.1	-1.5
5	BG	-1.4	-0.3	-1.4	0.1	0.6	-0.9	-2.1	-2.1	-1.6	1.3	-0.4	1.4	1.6	3.2	2.2	-0.7	-0.5	-1.3	0.1	0.6	-0.9	-1.9	-1.8	-1.4	1.7	1.9	1.4	0.2	-0.5	-0.1	3.8	1.1	-0.1	-0.6	0.2	-0.5	-1.7	-0.7	-0.4	-2.6	-1.5	-1.1	-2.6	-1.9	-1.3
6	CG	-1.4	-0.4	-1.5	0.0	0.5	-0.1	-1.2	-2.2	-1.6	1.2	-0.4	1.3	1.5	0.8	-0.5	-1.1	-0.5	-1.4	0.0	0.5	2.5	1.5	-1.5	-1.5	1.6	1.8	0.4	-0.6	-0.6	-0.2	3.8	1.0	-0.2	-0.7	0.1	-0.6	-1.7	-0.8	-1.0	-2.7	-1.6	-1.1	-2.7	-2.0	-1.4
7	DG	-1.2	-0.1	-1.2	0.3	0.8	-0.8	-1.9	-1.9	-1.4	1.5	-0.2	1.6	1.7	0.3	-0.8	-0.8	-0.3	-1.2	0.3	0.8	0.1	-0.9	-1.6	-1.2	1.9	2.1	3.8	2.9	0.0	0.1	4.0	1.3	0.0	-0.4	0.4	-0.3	-1.5	-0.6	-0.8	-2.5	-1.3	-1.8	-2.4	-1.7	-1.2
8	DD	-1.0	0.1	-1.0	0.5	1.0	-0.5	-1.7	-1.7	-1.1	1.7	0.0	1.9	2.0	0.5	-0.6	-0.6	0.0	-0.9	1.2	1.0	-0.5	-1.5	-1.4	-1.0	5.3	2.3	0.9	-0.1	-0.1	0.3	4.2	1.5	0.3	-0.2	0.6	-0.1	-1.3	-0.3	-0.5	-2.2	-1.1	-1.6	-2.2	-1.5	-0.9
9	BD	-1.1	0.0	-1.1	0.4	0.9	-0.6	-1.8	-1.8	-1.3	1.8	0.0	3.9	1.9	0.4	-0.7	-0.7	-0.2	-1.1	0.4	0.9	-0.6	-1.6	-1.5	-1.1	2.0	5.9	0.8	-0.3	-0.2	0.2	4.1	1.4	0.2	-0.3	0.5	-0.2	-1.4	-0.5	-0.6	-2.4	-1.2	-1.7	-2.3	-1.6	-1.0
10	BD	-1.0	0.1	-1.0	0.5	1.0	-0.5	-1.7	-1.7	-1.1	1.8	0.1	3.9	2.0	0.5	-0.6	-0.6	0.0	-0.9	0.5	1.0	-0.5	-1.5	-1.4	-1.0	2.1	2.3	0.9	-0.1	-0.1	0.3	4.2	1.5	0.3	-0.2	0.6	-0.1	-1.3	-0.3	-0.5	-2.2	-1.1	-1.6	-2.2	-1.5	-0.9
11	AD	-1.3	-0.2	-1.3	3.3	0.7	-0.8	-2.0	-2.0	-1.5	1.5	0.0	2.7	1.7	0.2	-0.9	-0.9	-0.4	-1.3	1.4	0.7	-0.8	-1.8	-1.7	-1.3	1.8	5.8	0.6	-0.5	-0.5	0.0	3.9	1.2	0.0	-0.5	0.3	-0.4	-1.6	-0.7	-0.9	-2.6	-1.4	-1.9	-2.5	-1.8	-1.2
12	HG	-1.2	-0.1	-1.2	0.3	0.8	1.3	0.0	-1.9	-1.4	1.5	-0.2	1.6	1.7	0.3	-0.8	-0.8	-0.3	-1.2	0.3	0.8	-0.7	-1.7	-1.6	-1.2	1.9	2.1	0.7	-0.4	-0.4	0.1	4.0	1.3	0.0	-0.4	0.4	-0.3	-1.5	2.1	-0.8	-2.5	1.2	-1.8	-1.7	-1.7	-1.2
13	HA	-0.6	-0.1	-1.2	0.3	0.8	-0.8	-1.9	2.0	0.5	1.5	-0.2	1.6	1.7	0.3	-0.8	-0.8	-0.3	-1.2	0.3	0.8	-0.7	-1.7	-1.4	-1.2	1.9	2.1	0.7	-0.4	-0.4	0.1	4.0	1.3	0.0	1.4	0.4	-0.3	-1.5	-0.6	-0.8	-2.5	-1.3	-1.8	-2.4	-1.7	-1.2
14	HA	-0.6	0.0	-1.1	0.4	0.9	-0.6	-1.8	1.9	0.4	1.6	-0.1	1.7	1.9	0.4	-0.7	-0.7	-0.2	-1.1	0.4	0.9	-0.6	-1.6	-1.3	-1.1	2.0	2.2	0.8	-0.3	-0.2	0.2	4.1	1.4	0.2	-0.3	0.5	-0.2	-1.4	-0.5	-0.6	-2.4	-1.2	-1.7	-2.3	-1.6	-1.0
15	HG	-1.3	-0.2	-1.3	0.2	0.7	-0.8	-1.2	-2.0	-1.5	1.4	-0.3	1.5	1.7	0.2	-0.9	-0.9	-0.4	-1.3	0.2	0.7	-0.8	-1.8	-1.7	-1.3	1.8	2.0	0.6	-0.5	-0.5	0.0	3.9	1.2	0.0	-0.5	0.3	-0.4	-1.6	2.3	0.7	-2.6	1.3	-0.1	-1.7	-1.8	-1.2
16	CD	-1.9	-0.8	-1.3	0.2	0.1	-1.4	-2.6	-2.6	-0.6	0.8	-0.5	0.9	1.1	-0.4	-1.5	-1.5	-1.0	0.4	2.4	2.0	1.5	-1.4	-2.3	1.4	1.2	1.4	1.6	0.1	-1.0	2.3	3.3	0.6	-0.6	-1.1	-0.3	-1.0	-2.2	-1.3	-0.1	-3.2	-2.0	-2.5	-3.1	-2.4	0.4
17	CH	-0.6	-0.5	0.6	-0.1	0.4	-0.2	-2.3	-0.9	-0.7	1.1	-0.6	1.1	1.3	-0.1	-1.2	-1.2	-0.7	-1.0	-0.1	0.4	-1.1	-2.2	1.3	-1.6	1.5	1.7	0.3	-0.8	-0.8	-0.3	3.6	0.9	-0.4	-0.8	0.0	-0.7	-1.9	1.3	-1.2	-2.9	-0.8	-2.2	-2.8	0.8	-1.6
18	CA	0.3	-0.3	1.9	0.1	0.6	1.7	-0.7	-2.1	1.2	1.3	-0.4	1.4	1.6	0.1	-1.0	-1.0	-0.5	-1.3	0.1	1.8	-0.9	-1.9	-1.8	-1.0	1.7	1.9	0.5	-0.5	-0.5	-0.1	3.8	1.1	-0.1	-0.6	0.2	-0.5	-1.7	-0.7	-0.9	-2.6	-1.5	-2.0	-2.6	-1.9	-1.3
19	BD	-1.8	-0.7	0.5	1.1	0.2	-1.4	-2.6	-2.6	-0.6	1.1	0.8	2.5	2.7	0.1	-1.4	-1.5	2.0	0.1	2.5	0.1	-0.4	-2.4	-2.3	0.1	1.2	1.4	0.0	-1.0	-1.0	0.2	3.4	0.7	-0.6	-1.1	-0.2	-1.0	-2.1	-1.2	-1.4	-3.1	-2.0	-2.4	-3.0	-2.4	-1.8
20	BD	-1.8	-0.7	-1.8	-0.3	0.2	-1.3	-2.5	-2.5	-2.0	1.2	1.7	2.9	1.2	-0.3	-1.4	-1.4	1.8	-1.7	-0.3	0.2	-0.7	-1.4	-2.2	-1.8	1.3	1.5	2.7	0.1	-0.9	1.8	3.4	0.7	2.4	-1.0	-0.2	0.8	-2.1	-1.2	0.8	-3.0	-1.9	0.4	-3.0	-2.3	-1.7
21	BA	-1.3	2.6	-1.3	0.2	0.7	1.8	0.5	-2.0	0.4	1.4	-0.1	1.5	1.7	0.2	-0.9	-0.9	-0.4	-1.3	0.2	0.7	-0.8	-1.8	-1.7	-1.3	1.8	2.0	0.6	-0.5	-0.5	0.0	3.9	1.2	0.0	-0.5	2.0	-0.4	-1.6	-0.7	-0.9	-2.6	-1.4	-1.9	-2.5	-1.8	-1.2
22	BA	-1.2	2.5	-1.2	0.3	0.8	1.7	0.3	-1.9	0.4	1.5	-0.2	1.6	1.7	0.3	-0.8	-0.8	-0.3	-1.2	0.3	0.8	-0.7	-1.3	-1.6	-1.2	1.9	2.1	0.7	-0.4	-0.4	0.1	4.0	1.3	0.0	-0.4	0.4	-0.3	-1.5	-0.6	-0.8	-2.5	-1.3	-1.8	-2.4	-1.7	-1.2
23	BI	-1.3	-0.2	-1.3	0.2	0.7	-0.8	-2.0	-2.0	-1.5	1.4	-0.3	1.5	1.7	0.2	-0.4	-0.9	2.7	-1.3	0.2	0.7	-0.8	-1.8	-1.7	-1.3	1.8	2.0	0.6	-0.5	-0.5	0.0	3.9	1.2	0.0	-0.5	0.3	-0.4	-0.7	-0.7	1.8	-2.6	-1.4	0.5	-2.5	-1.8	1.2
24	DD	-1.0	0.1	-1.0	0.5	1.0	-0.5	-1.7	-1.7	-1.1	1.7	0.0	1.9	2.0	0.5	-0.6	-0.6	0.0	-0.9	0.6	1.0	-0.5	-1.5	-1.4	-1.0	5.3	2.3	0.9	-0.1	-0.1	0.3	4.2	1.5	0.3	-0.2	0.6	-0.1	-1.3	-0.3	-0.5	-2.2	-1.1	-1.6	-2.2	-1.5	-0.9
25	FF	-1.2	-0.1	-1.2	0.3	0.8	1.5	-1.9	-1.9	-1.4	1.5	-0.2	1.6	1.7	0.5	-0.8	-0.8	-0.3	-1.2	0.3	0.8	2.0	-1.7	-1.6	-1.2	1.9	2.1	0.7	-0.4	-0.4	0.1	4.0	1.3	0.0	-0.4	0.4	3.1	0.4	-0.6	-0.8	-2.5	-1.3	-1.8	-2.4	-1.7	-1.2
26	BI	-1.1	0.0	-1.1	0.4	0.9	-0.6	-1.8	-1.8	-1.3	1.6	-0.1	1.7	1.9	0.4	-0.4	-0.7	2.9	-1.1	0.4	0.9	-0.6	-1.6	-1.5	-1.1	2.0	2.2	1.0	-0.3	-0.2	1.4	4.1	1.4	0.2	-0.3	0.5	-0.2	-1.4	-0.5	-0.6	-2.4	-1.2	-1.7	-2.3	-1.6	-1.0
27	BD	-1.1	0.0	-1.1	0.4	0.9	-0.6	-1.8	-1.8	-1.3	1.8	0.0	4.0	1.9	0.4	-0.7	-0.7	-0.2	-1.1	0.4	0.9	-0.6	-1.6	-1.5	-1.1	3.3	2.2	0.8	-0.3	-0.2	0.2	4.1	1.4	0.2	-0.3	0.5	-0.2	-1.4	-0.5	-0.6	-2.4	-1.2	-1.7	-2.3	-1.6	-1.0
28	BF	-1.4	1.5	-1.5	0.9	0.5	-1.0	-2.2	-2.2	-1.6	1.4	2.1	1.3	1.5	2.6	-0.1	-1.1	-0.5	-1.4	1.0	0.5	-0.9	-2.0	-1.9	-1.5	1.6	1.8	0.8	-0.6	-0.6	-0.2	3.8	1.0	-0.2	-0.7	0.1	-0.6	-1.7	-0.8	-1.0	-2.7	-1.6	-2.1	-2.7	-2.0	-1.4
29	AC	-1.2	-0.1	1.6	0.3	0.8	-0.8	-1.9	-1.9	-1.4	1.5	0.5	1.6	1.7	0.3	-0.8	-0.8	-0.3	-1.2	0.3	1.9	-0.7	-1.7	-1.0	1.5	1.9	2.1	0.7	-0.4	-0.4	0.1	4.0	1.3	0.0	-0.4	0.4	-0.3	-1.5	-0.6	-0.8	-2.5	-1.3	-1.8	-2.4	-1.7	-1.2
30	BA	-1.2	2.3	-1.2	0.6	0.8	-0.8	-1.9	-1.9	-1.4	1.5	1.9	1.6	1.7	1.4	-0.8	-0.8	0.3	-1.2	0.3	0.8	-0.7	-1.7	-1.6	-1.2	1.9	2.1	0.7	-0.4	-0.4	0.1	4.0	1.3	0.0	-0.4	0.4	-0.3	-1.5	-0.6	-0.8	-2.5	-1.3	-1.8	-2.4	-1.7	-1.2
31	BC	-0.8	0.3	-0.9	0.6	1.2	-0.4	-1.6	-1.6	-1.0	1.8	2.2	1.9	2.1	0.6	-0.5	-0.5	0.1	-0.8	0.8	1.1	-0.3	-1.4	-1.3	-0.8	2.2	2.4	1.0	0.0	0.0	0.5	4.4	1.7	0.4	-0.1	0.7	0.0	-1.1	-0.2	-0.4	-2.1	-1.0	-1.4	-2.0	-1.4	-0.8
32	BB	-0.8	0.3	-0.9	0.6	1.2	-0.4	-1.6	-1.6	-1.0	3.6	0.2	2.0	2.1	0.6	-0.5	-0.5	0.1	-0.8	0.7	1.1	-0.3	-1.4	-1.3	-0.8	2.2	2.4	1.0	0.0	0.0	0.5	4.4	1.7	0.4	-0.1	0.7	0.0	-1.1	-0.2	-0.4	-2.1	-1.0	-1.4	-2.0	-1.4	-0.8
33	BA	-0.8	2.5	-0.9	0.8	1.2	-0.4	-1.6	-1.6	-1.0	1.8	0.2	1.9	2.1	0.6	-0.5	-0.5	0.1	-0.8	0.7	1.1	-0.3	-1.4	-1.3	-0.8	2.2	2.4	1.0	0.0	0.0	0.5	4.4	1.7	0.4	-0.1	0.7	0.0	-1.1	-0.2	-0.4	-2.1	-1.0	-1.4	-2.0	-1.4	-0.8
34	BI	-1.0	0.1	-1.0	0.5	1.0	-0.5	-1.7	-1.7	-1.1	1.7	0.0	1.8	2.0	0.7	-0.6	-0.6	2.8	-0.9	0.5	1.0	-0.5	-1.5	-1.4	-1.0	2.1	2.3	0.9	-0.1	-0.1	0.6	4.2	1.5	0.3	-0.2	0.6	-0.1	-1.3	-0.3	-0.5	-2.2	-1.1	-1.6	-2.2	-1.5	-0.9
35	BF	-1.2	1.6	-1.2	0.3	0.8	-0.8	-1.9	-1.9	-1.4	1.6	2.0	1.6	1.7	2.4	-0.8	-0.8	-0.3	-1.2	0.3	0.8	-0.7	-1.7	-1.6	-1.2	1.9	2.1	1.0	-0.4	-0.4	0.1	4.0	1.3	0.0	-0.4	0.4	-0.3	-1.5	-0.6	-0.8	-2.5	-1.3	-1.8	-2.4	-1.7	-1.2
36	FC	-0.8	0.3	-0.9	0.6	1.2	-0.4	-1.6	-1.6	-1.0	1.8	0.2	1.9	2.1	0.6	-0.5	-0.5	0.1	-0.8	0.7	1.1	2.2	-1.4	-1.3	-0.3	2.2	2.4	1.0	0.0	0.0	0.5	4.4	1.7	0.4	-0.1	0.7	0.0	-1.1	-0.2	-0.4	-2.1	-1.0	-1.4	-2.0	-1.4	-0.8
37	FB	-1.0	0.1	-1.0	0.5	1.0	-0.5	-1.7	-1.7	-1.1	1.7	0.0	1.8	2.0	3.2	-0.6	-0.6	0.6	-0.9	0.5	1.0	-0.5	-1.5	-1.4	-1.0	2.1	2.3	1.2	-0.1	-0.1	0.3	4.2	1.5	0.3	-0.2	0.6	-0.1	-1.3	-0.3	-0.5	-2.2	-1.1	-1.6	-2.2	-1.5	-0.9
38	FF	-1.4	-0.3	-1.4	0.1	0.6	1.7	-2.1	-2.1	-0.8	1.3	-0.4	1.4	1.6	0.5	-1.0	-1.0	-0.5	-1.3	0.1	0.6	2.2	-1.9	-1.8	-0.5	1.7	1.9	0.5	-0.5	-0.5	-0.1	3.8	1.1	-0.1	-0.6	0.2	3.1	-1.7	-0.7	-0.3	-2.6	-1.5	-2.0	-2.6	-1.9	-1.3
39	IB	-1.1	0.0	-1.1	0.4	0.9	-0.6	-1.8	-1.8	-1.3	1.6	-0.1	1.7	1.9	0.9	-0.7	0.1	2.9	-1.1	0.4	0.9	-0.6	-1.6	-1.5	-1.1	2.0	2.2	0.8	-0.3	-0.2	0.6	4.1	1.4	0.2	-0.3	0.5	-0.2	-1.4	-0.5	-0.6	-2.4	-1.2	-1.7	-2.3	-1.6	-1.0
40	IF	-1.5	-0.4	-1.6	-0.1	0.5	-0.4	-2.3	-2.3	1.3	1.1	-0.5	1.2	1.4	-0.1	-1.1	-1.2	-0.1	-1.5	0.0	0.4	-0.5	-2.1	-1.1	1.9	1.6	1.7	0.3	-0.7	-0.7	-0.2	3.7	1.0	-0.3	-0.8	0.1	1.4	-1.8	-0.1	1.9	-2.8	-1.7	-2.1	-2.7	-2.1	-1.5
41	IH	-1.6	-0.5	-1.7	-0.1	0.4	-1.2	-2.3	-2.3	-0.4	1.1	-0.6	1.1	1.3	-0.1	-1.2	-1.2	-0.7	-1.6	-0.1	0.4	0.1	-2.2	-1.4	1.6	1.5	1.7	0.3	-0.8	-0.8	-0.3	3.6	0.9	-0.4	-0.8	0.0	0.5	-1.9	0.1	1.3	-2.9	-1.7	-2.2	-1.4	1.0	2.2
42	IA	-1.4	-0.4	-1.5	0.0	0.5	-0.4	-2.2	-1.0	1.9	1.2	-0.4	1.3	1.5	0.7	-1.1	-0.3	1.6	-1.4	0.0	0.5	-0.1	-2.0	-1.9	1.6	1.6	1.8	0.4	-0.6	-0.6	-0.2	3.8	1.0	-0.2	-0.7	0.1	-0.6	-1.7	-0.8	-1.0	-2.7	-1.6	-2.1	-2.7	-2.0	-1.4
43	ID	-1.1	0.0	-1.1	0.4	0.9	-0.6	-1.8	-1.8	-1.3	1.6	-0.1	1.7	1.9	0.4	-0.7	-0.7	-0.2	-1.1	0.4	0.9	-0.6	-1.6	-1.2	-1.1	2.0	2.2	1.8	-0.3	0.5	3.6	4.1	1.4	0.2	-0.3	0.5	-0.2	-1.4	-0.5	-0.6	-2.4	-1.2	-1.7	-2.3	-1.6	-1.0
44	DB	-0.8	0.3	-0.9	0.6	1.2	-0.4	-1.6	-1.6	-1.0	1.8	0.2	3.7	2.1	0.6	-0.5	-0.5	0.1	-0.8	0.7	1.1	-0.3	-1.4	-1.3	-0.8	2.5	2.4	1.0	0.0	0.0	0.5	4.4	1.7	0.4	-0.1	0.7	0.0	-1.1	-0.2	-0.4	-2.1	-1.0	-1.4	-2.0	-1.4	-0.8
45	DA	-1.0	0.1	-1.0	2.6	1.0	-0.5	-1.7	-1.7	-1.1	1.7	0.0	2.8	2.0	0.5	-0.6	-0.6	0.0	-0.9	2.4	1.0	-0.5	-1.5	-1.4	-1.0	2.1	2.3	0.9	-0.1	-0.1	0.3	4.2	1.5	0.3	-0.2	0.6	-0.1	-1.3	-0.3	-0.5	-2.2	-1.1	-1.6	-2.2	-1.5	-0.9
46	DB	-0.8	0.3	-0.9	0.6	1.2	-0.4	-1.6	-1.6	-1.0	1.8	0.2	2.6	2.1	0.6	-0.5	-0.5	0.1	-0.8	0.7	1.1	-0.3	-1.4	-1.3	-0.8	2.2	2.4	3.6	0.0	0.0	0.5	4.4	1.7	0.4	-0.1	0.7	0.0	-1.1	-0.2	-0.4	-2.1	-1.0	-1.4	-2.0	-1.4	-0.8
47	AC	-0.7	0.4	1.3	0.8	1.3	-0.2	-1.4	-1.4	-0.9	2.0	0.3	2.1	2.3	0.8	-0.3	-0.3	0.2	-0.6	0.8	1.3	-0.2	-1.2	-1.1	-0.7	2.4	2.6	1.2	0.1	0.2	0.6	4.5	1.8	0.6	0.1	0.9	0.2	-1.0	-0.1	-0.2	-1.9	-0.8	-1.3	-1.9	-1.2	-0.6

Supplementary Figure S2 - The pairPSSM of protein complex 1a2kAD. There are 47 pairs of contact residues.

Each row represents 45 types of energy in a specific contact position (9 clusters in 20 amino acids result in 45 types cluster pair). The abbreviation A represent residue [Ala and Gly]; B: [Val, Met, Leu and Ile]; C: [Pro, Ser and Thr]; D: [Phe, Tyr and Trp]; E: [Cys]; F: [His and Arg]; G: [H]: [Asp and Glu]; I: [Asn and Gln].

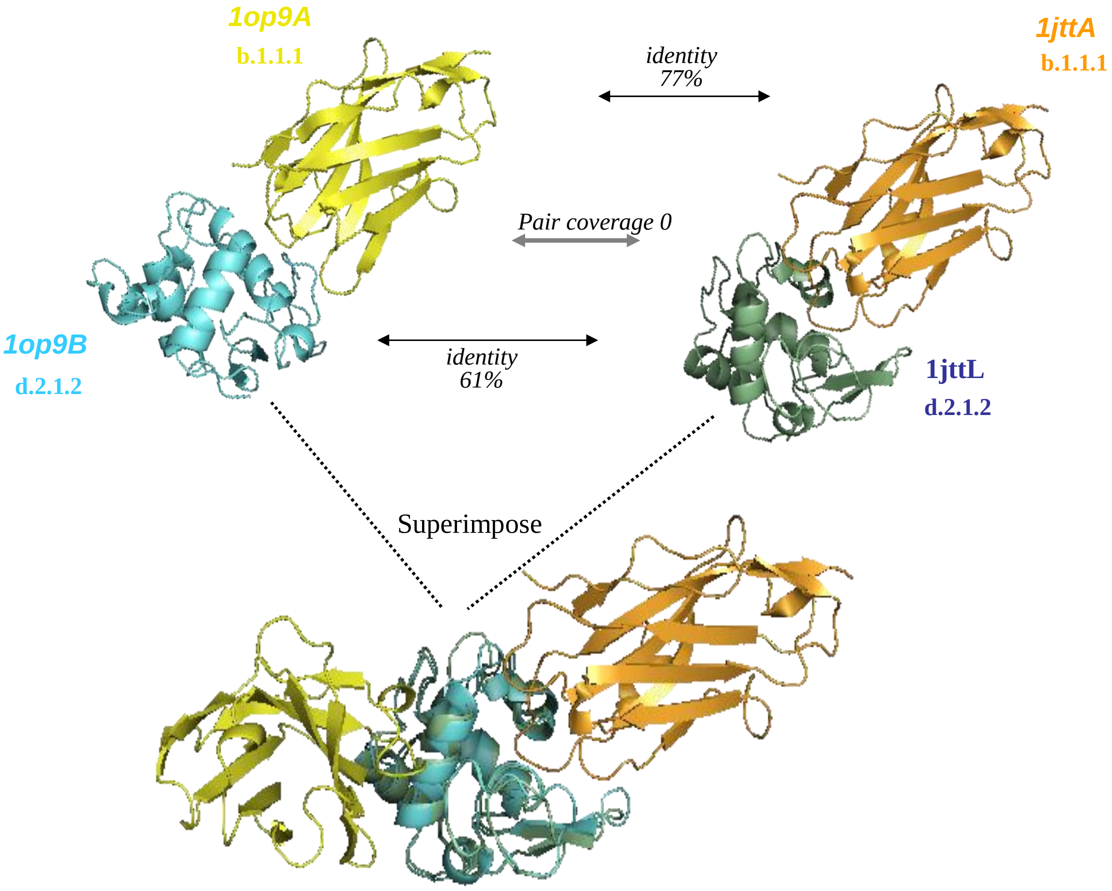

Supplementary Figure S3 - The interactive types of two hydrolase-antibody complexes 1op9AB and 1jttAL.

We superimpose the 1op9B and 1jttL and discover the binding site of the two proteins at two different sites (bottom). 1op9 (PDB ID) is a hydrolase-antibody complex in human and 1jtt (PDB ID) also is a hydrolase-antibody in chicken. The A chain of 1op9 and A chain of 1jtt both contain the V set antibody variable domain (SCOP id, b.1.1.1) and the sequence identity between the two proteins is as high as 77%. The B chain of 1op9 and B chain of 1jtt both contain the C-type lysozyme domain (SCOP id, d.2.1.2) and the sequence identity between the two proteins is as high as 61%. By study the interactive site of the two complexes, we discover both the binding area of 1op9A and 1jttA at the variable region of V set antibody variable domain. However, the binding sites of 1op9B and 1jttL are very different. We superimpose the 1op9B and 1jttL and discover the binding site of the two proteins at two different sides. Therefore, the pair coverage between the two complexes is very low.

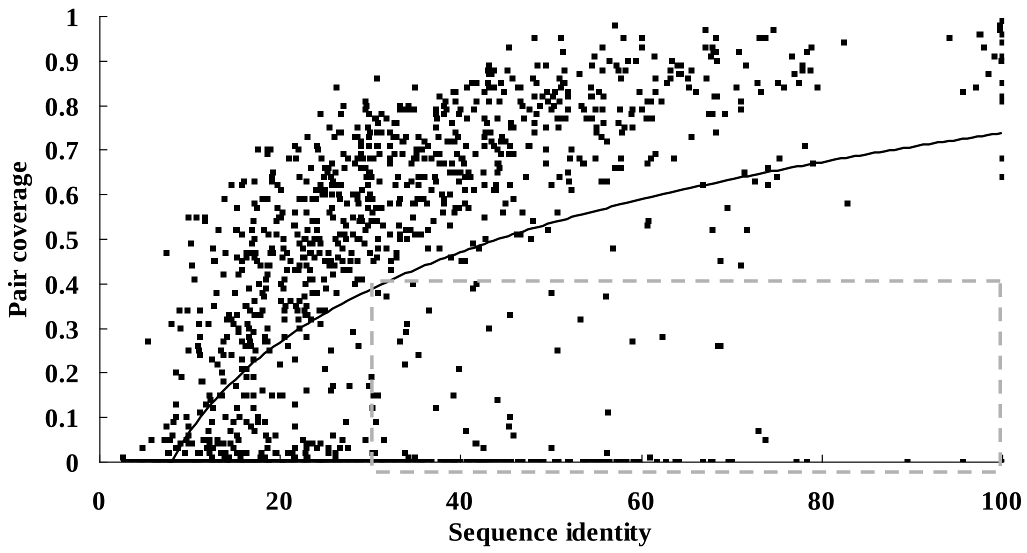

Supplementary Figure S4 - The relationship between sequence identity and pair coverage of 1412 pairs of related homo dimers.

The dots in gray box are the exceptions of the pairs of dimer with > 30% sequence identity but pair coverage < 0.4.

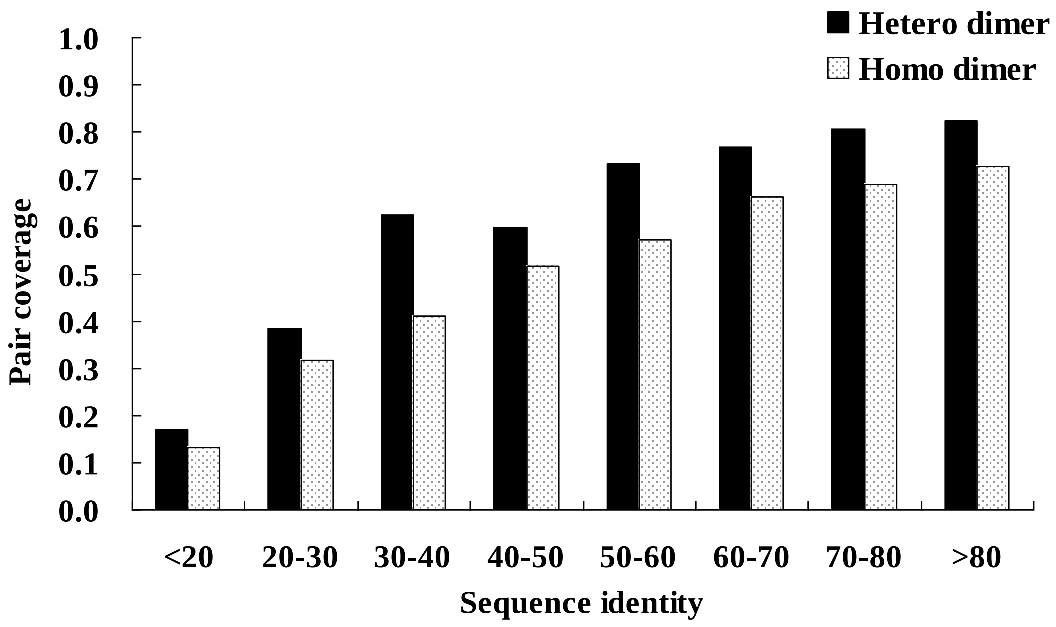

Supplementary Figure S5 - The average pair coverage in different sequence identity interval.

The black and gray bars are for heterodimers and homodimers, respectively.

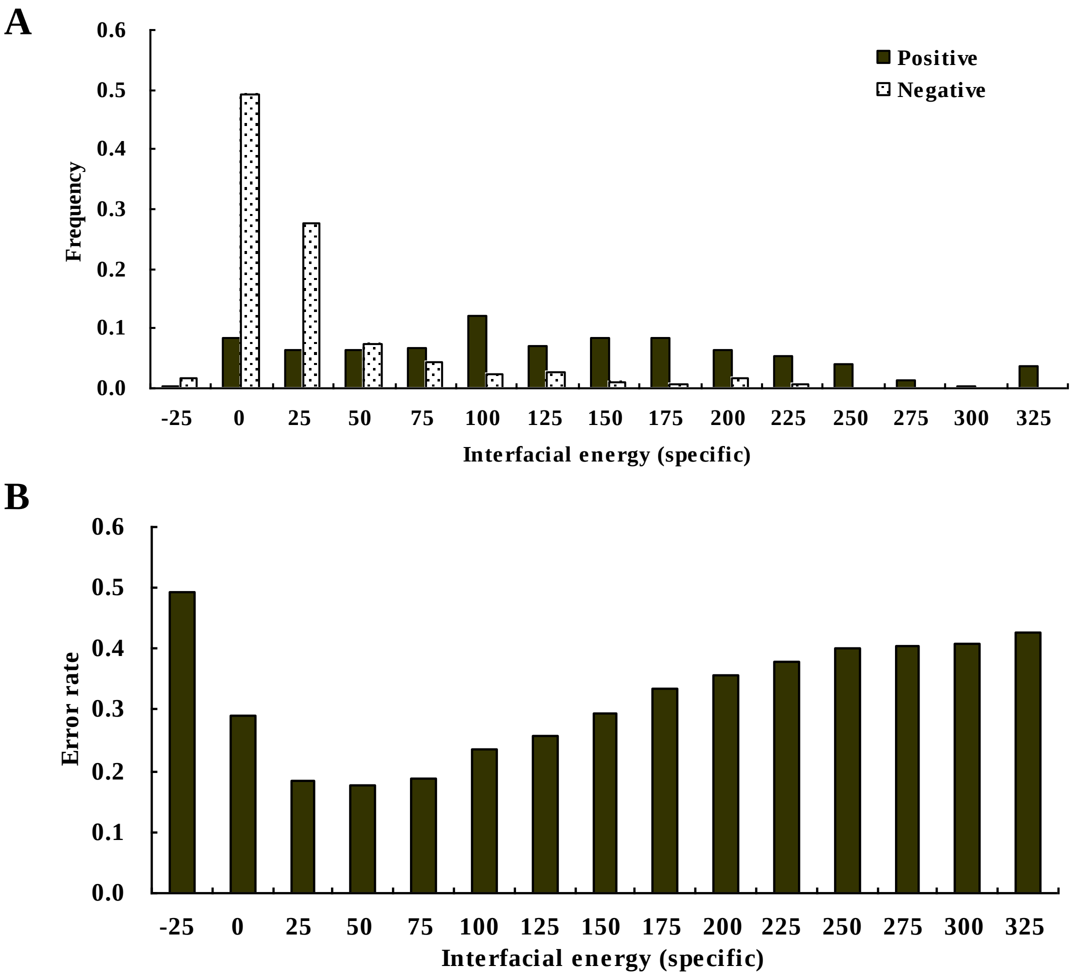

Supplementary Figure S6 - Determining the threshold of specific interfacial energy on distinguishing the true protein complex and unreasonable protein pairs.

The specific interfacial energy is calculated from *pair*PSSM. (A) The frequency of positives and negatives in different interfacial energy intervals. (B) The error rate of prediction at different thresholds. A threshold of 50 is consequently set from this histogram.

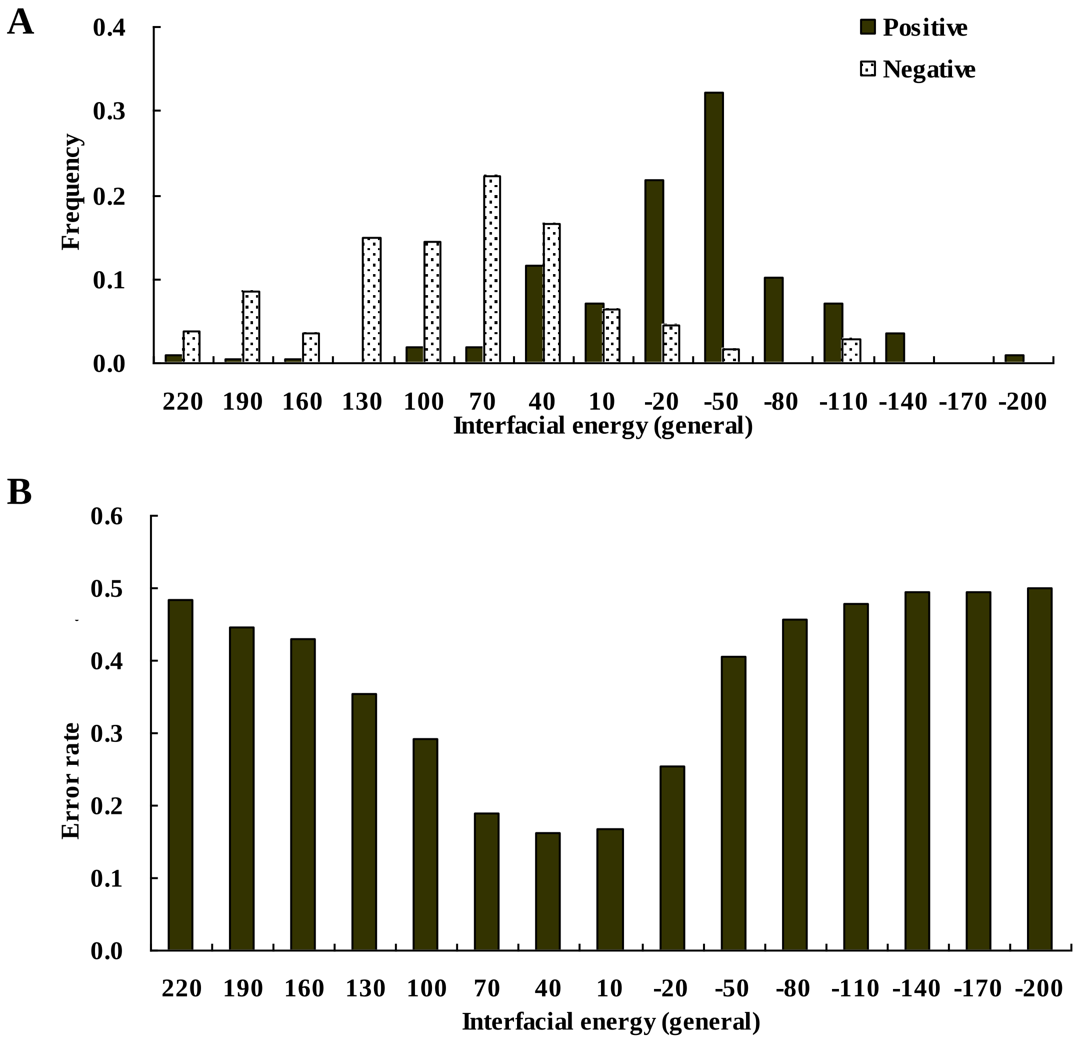

Supplementary Figure S7 - Determining the threshold of general interfacial energy on distinguishing the true protein complex and unreasonable protein pairs.

The general interfacial energy is calculated from general empirical matrix. (A) The frequency of positives and negatives in different interfacial energy intervals. (B) The error rate of prediction at different thresholds. A threshold of 10 is consequently set from this histogram.

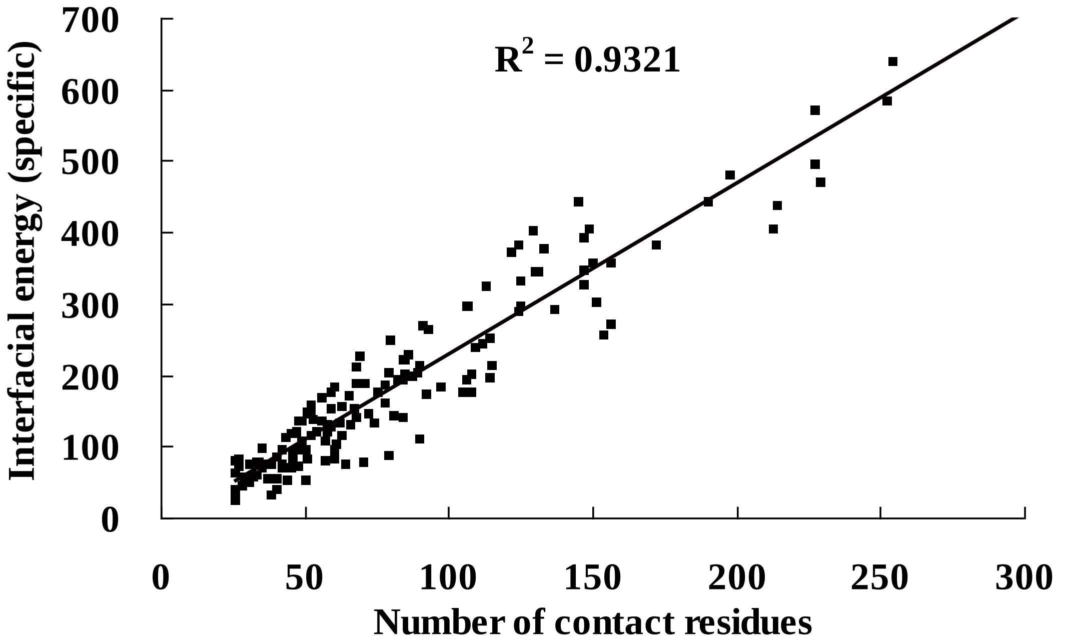

Supplementary Figure S8 - The relationship between number of contact residues in 3D-dimers and its specific interfacial energies which are calculated from *pair*PSSM.

The correlation coefficient is 0.9321.

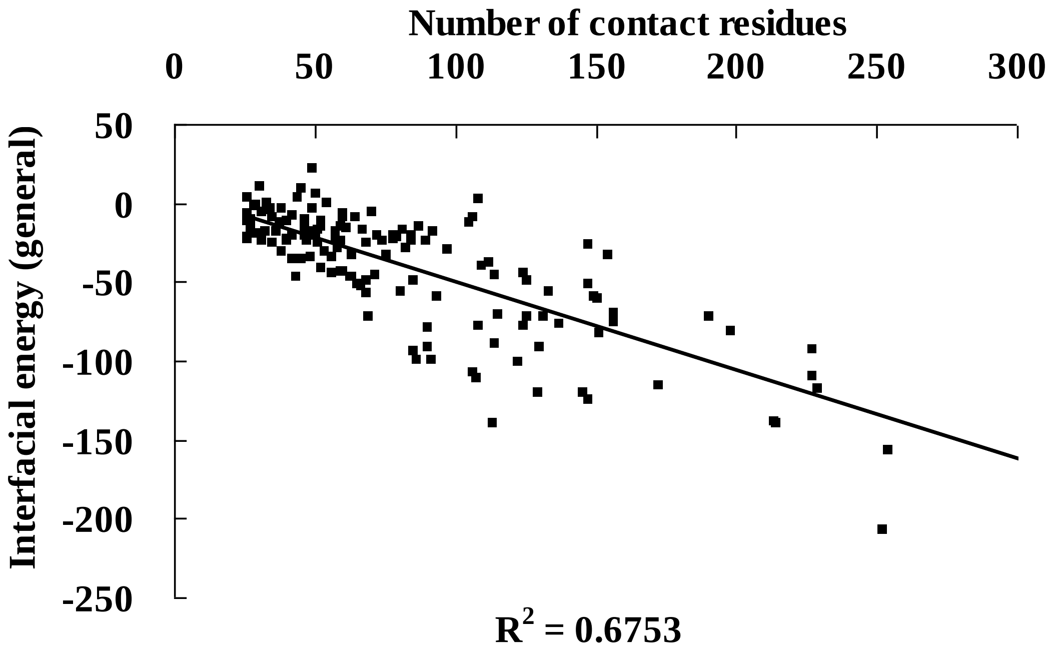

Supplementary Figure S9 - The relationship between number of contact residues in 3D-dimer and its general interfacial energies with are calculated from general empirical matrix.
The correlation coefficient is 0.6753.

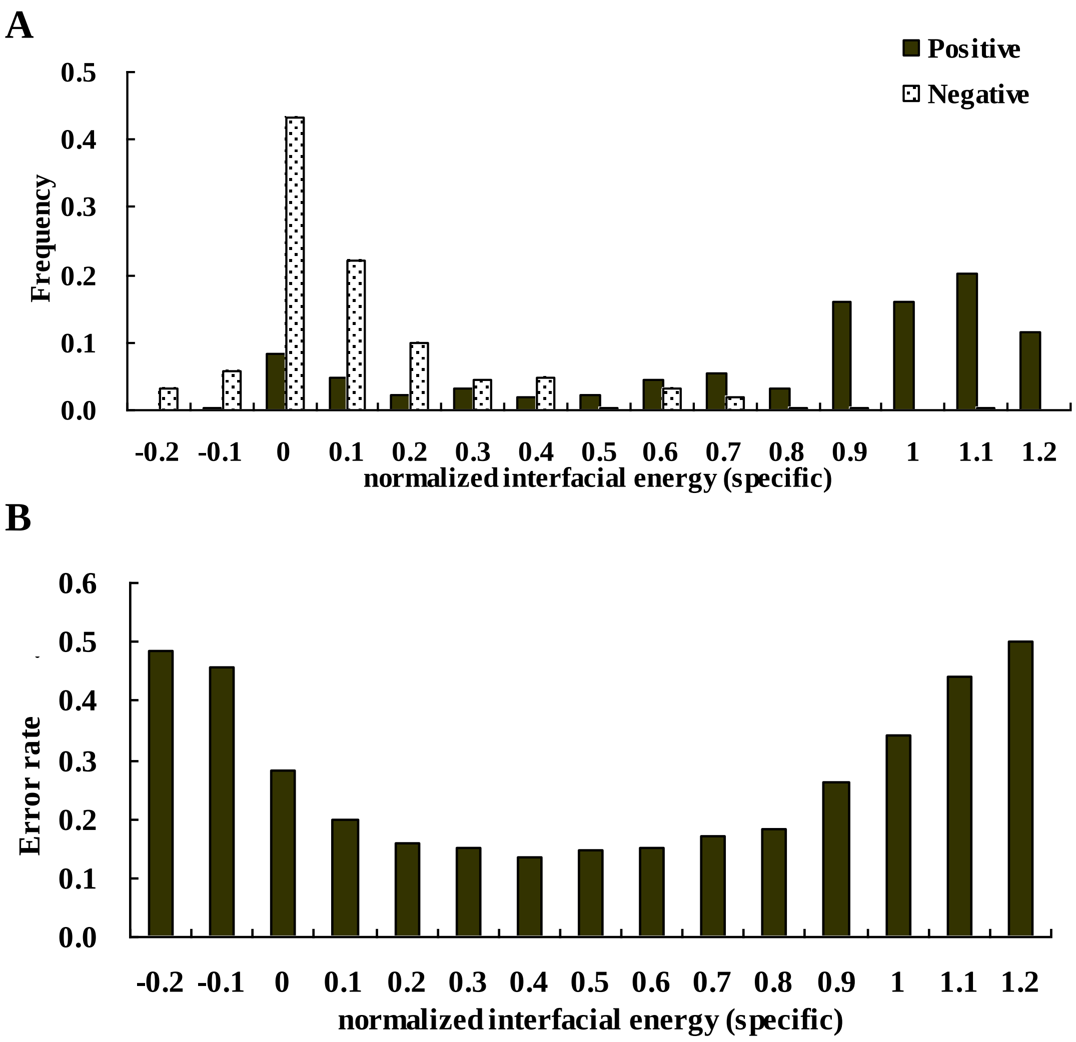

Supplementary Figure S10 - Determining the threshold of normalized specific interfacial energy on distinguishing the true protein complex and unreasonable protein pairs.

The method to calculate normalized specific interfacial energy describes in text. (A) The frequency of positives and negatives in different interfacial energy intervals. (B) The error rate of prediction at different thresholds. A threshold of 0.4 is consequently set from this histogram.

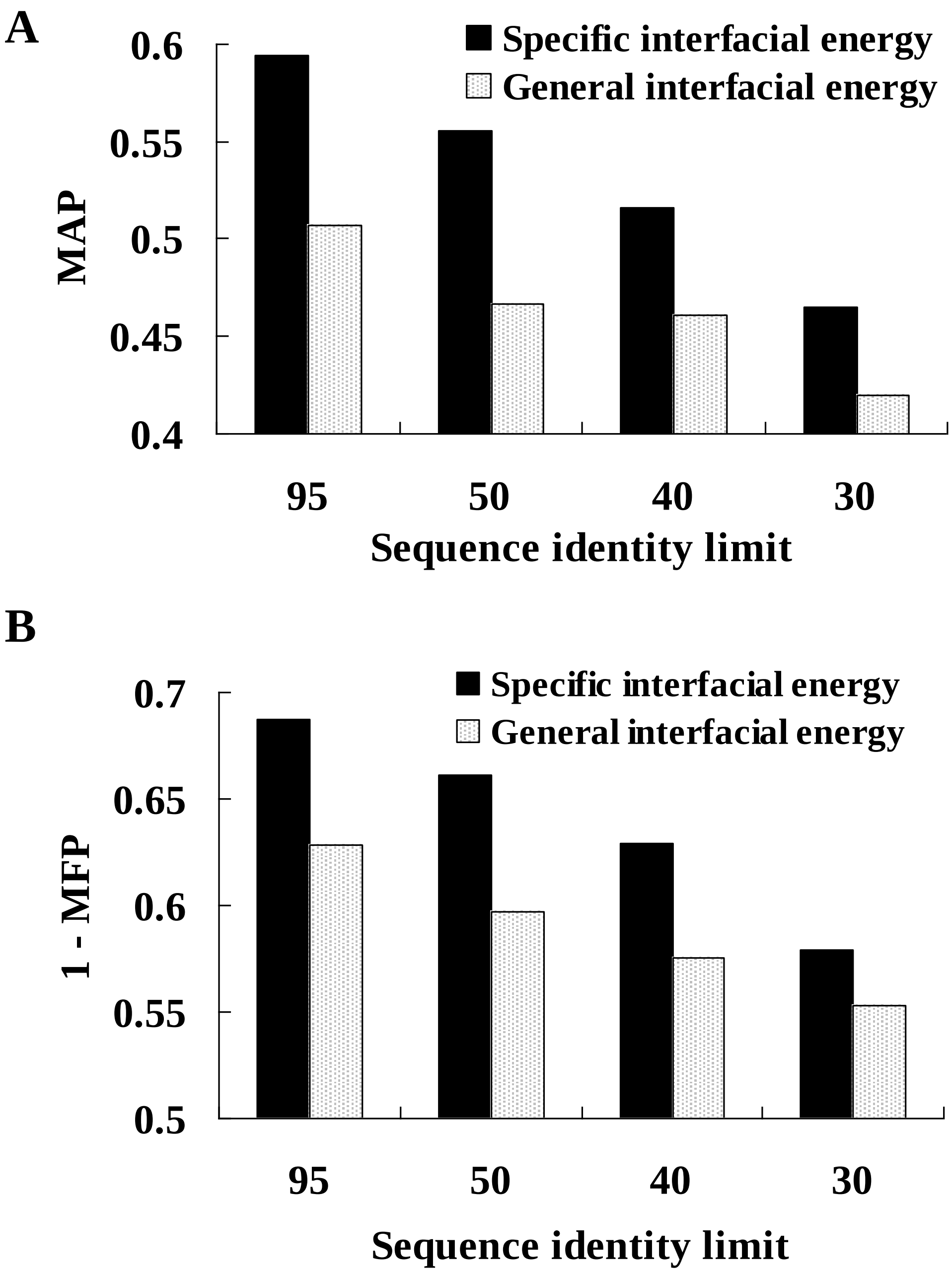

Supplementary Figure S11 - The mean average positions and mean false positive rate of 101 queries (the unannotated candidates are removed).
The (A) MAP and (B) 1-MFP distributions between specific interfacial energy and general interfacial energy in sequence identity limit with 95%, 50%, 40% and 30%. Sequence identity limit means that if one protein of candidate with sequence identity > sequence identity limit, the candidate is removed.

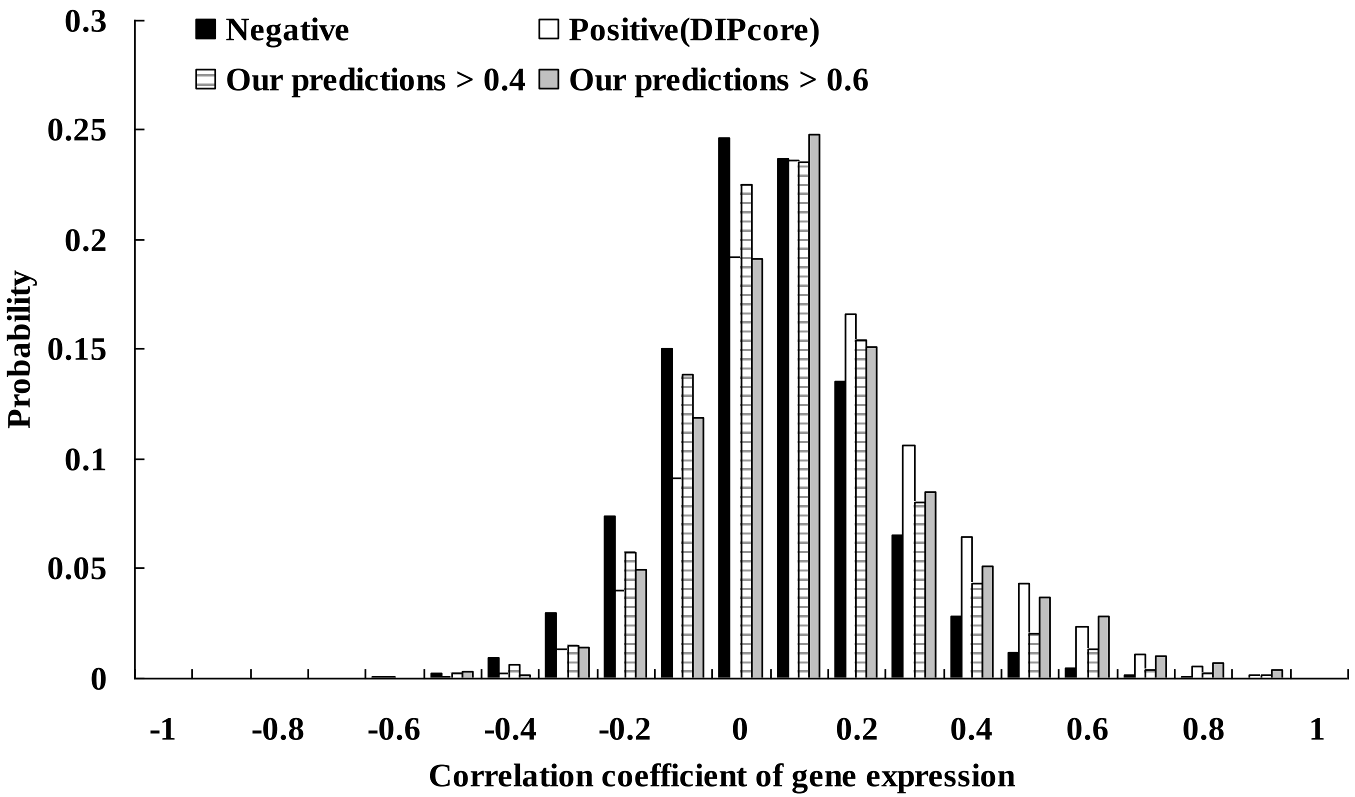

Supplementary Figure S12 - Distributions of the correlation coefficients of gene expression profiles for four interacting protein pairs sets.

We identified homologous PPIs with thresholds 0.4 (band) and 0.6 (gray), the DIP core set (white), and the non-interacting protein pairs (black). The correlations of our identified homologous PPIs are much higher than the one of non-interacting protein pairs.

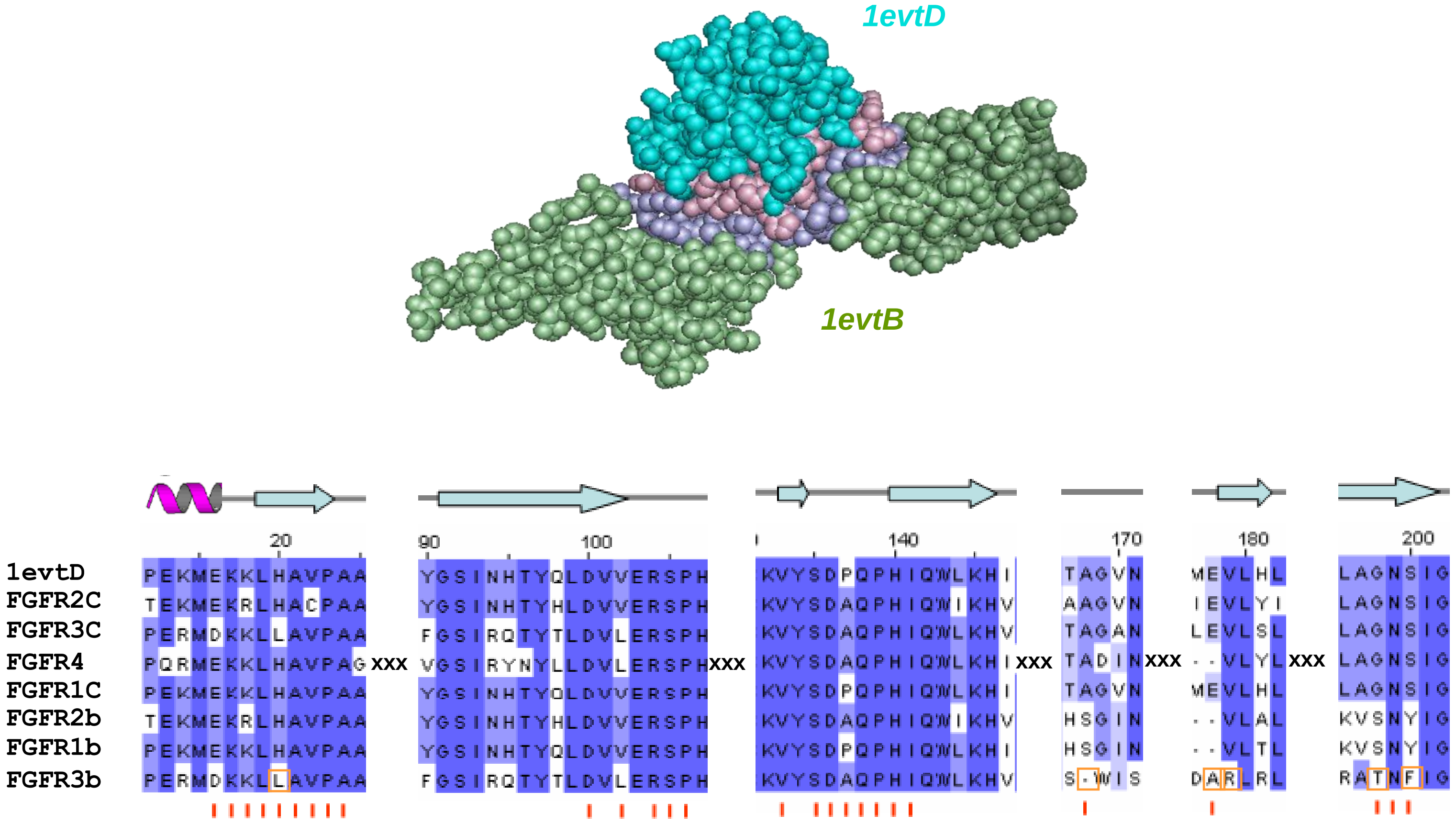

Supplementary Figure S13 - The 3D-structure of 1evtBD and multiple sequence alignment of seven homologous FGF receptors.
(A) The 3D-structure of 1evtBD. (B) The multiple sequence alignment of seven homologous FGF receptors to chain D of 1evt. The red bars in the bottom are the contact positions in 1evt D chain.

Supplementary Table S1 - The frequency of amino acid occurs in protein surface and whole protein. The data of whole protein is downloaded from SWISSPROT database.

Amino acid	Surface (%)	Whole protein (%)
ILE (I)	2.0	5.9
VAL (V)	2.8	6.7
LEU (L)	3.3	9.6
PHE (F)	1.7	4.0
CYS (C)	0.3	1.5
MET (M)	1.3	2.4
ALA (A)	6.2	7.8
GLA (G)	7.5	6.9
THR (T)	6.1	5.4
SER (S)	7.0	6.8
TRP (W)	0.5	1.1
TYR (Y)	2.1	3.1
PRO (P)	6.0	4.8
HIS (H)	2.2	2.3
ASN (N)	6.7	4.2
GLN (Q)	5.9	4.0
ASP (D)	9.6	5.3
GLU (E)	11.7	6.7
LYS (K)	10.7	5.9
ARG (R)	6.2	5.4

Supplementary Table S2 - The 24 pairs of related heterodimers with > 30% sequence identity but with pair coverage < 0.4.

Template	Related dimer	Pair coverage	IdeA[a]	IdeB[b]	DomainA[c]	DomainB[d]
1kxqBG	1kxtEF	0.00	100.0	69.4	c.1.8.1	b.1.1.1
1kxqBG	1kxvAC	0.00	100.0	62.1	c.1.8.1	b.1.1.1
1op9AB	1jtpAL	0.00	76.7	58.9	b.1.1.1	d.2.1.2
1ewyAC	1gaqAB	0.00	52.4	70.2	b.43.4.2, c.25.1.1	d.15.4.1
1op9AB	1p2cBC	0.00	49.1	60.5	b.1.1.1	d.2.1.2
1op9AB	1j1oHY	0.00	47.3	60.5	b.1.1.1	d.2.1.2
1jb0AD	1jb0BD	0.00	47.3	100.0	f.29.1.1	d.187.1.1
1jb0AC	1jb0BC	0.00	47.3	100.0	f.29.1.1	d.58.1.2
1jb0AF	1jb0BF	0.00	47.3	100.0	f.29.1.1	f.23.16.1
1op9AB	1bvkBC	0.00	46.4	60.5	b.1.1.1	d.2.1.2
1cl7HL	1deeAD	0.00	42.2	60.2	b.1.1.1	b.1.1.1, b.1.1.2
1k3zAD	1iknCD	0.00	54.0	38.3	b.1.18.1	d.211.1.1
1op3HK	1uweLV	0.00	35.7	54.9	b.1.1.2	b.1.1.2
1bzqAL	1h0dBC	0.00	34.6	66.4	d.5.1.1	b.1.1.1
1mdaHM	2bbkJM	0.00	31.4	77.7	b.69.2.1	g.21.1.1
1dxrCL	1eysCM	0.02	48.2	32.0	a.138.1.2	f.26.1.1
1bqhAG	1bqhDK	0.03	100.0	100.0	b.1.1.2	b.1.1.1
1hezAE	1hezCE	0.03	100.0	100.0	b.1.1.1	d.15.7.1
1s6bAB	1oqsAB	0.05	45.6	43.5	a.133.1.2	a.133.1.2
1op9AB	1fbiHX	0.12	47.9	56.6	b.1.1.1	d.2.1.2
1bd2AD	1mi5AD	0.14	84.3	56.0	d.19.1.1	b.1.1.1
1r8sAE	1re0AB	0.30	81.5	37.7	c.37.1.8	a.118.3.1
1abrAB	1m2tAB	0.38	40.3	52.7	d.165.1.1	b.42.2.1, b.42.2.1
1hcfAX	1wwwWX	0.39	51.4	44.4	g.17.1.3	b.1.1.4

[a] The sequence identity between the first chain of template and the first chain of protein of the related dimer.
[b] The sequence identity between the second chain of template and the second chain of protein of the related dimer.
[c] The interacting domains in the first chain of template.
[d] The interacting domains in the second chain of template.

Supplementary Table S3 - **Average precisions and false positive rates of specific interfacial energy and general interfacial energy on 182 queries. The unannotated candidates are considered as negative.**

Query #	PDB ID	No. of Candidates	No. of Positives	No. of Negatives	AP (specific)	FP (specific)	AP (general)	FP (general)
1	1a0rBP	515	2	513	0.83	0.00	0.20	0.01
2	1a2kAD	74	2	72	0.25	0.06	0.03	0.66
3	1a6dAB	66	1	65	0.07	0.20	0.07	0.20
4	1a9nAB	264	2	262	0.53	0.05	0.51	0.23
5	1agrDH	18	1	17	0.50	0.06	0.08	0.71
6	1aisAB	4	2	2	0.58	0.50	0.50	0.75
7	1auiAB	117	5	112	0.81	0.19	0.58	0.22
8	1b34AB	10	5	5	0.51	0.52	0.61	0.52
9	1b7tAZ	30	8	22	0.73	0.16	0.30	0.47
10	1bi8AB	2200	6	2194	0.00	0.44	0.00	0.66
11	1buhAB	110	1	109	1.00	0.00	0.05	0.17
12	1c9bMN	6	2	4	0.83	0.13	0.75	0.25
13	1d3bEF	36	11	25	0.57	0.26	0.36	0.38
14	1dceAB	9	3	6	0.57	0.50	0.63	0.33
15	1dkgAD	15	1	14	0.50	0.07	0.50	0.07
16	1dn1AB	28	4	24	0.25	0.43	0.42	0.38
17	1doaAB	37	3	34	1.00	0.00	0.30	0.13
18	1e79AG	4	2	2	0.83	0.25	1.00	0.00
19	1eesAB	105	3	102	0.28	0.05	0.53	0.11
20	1eqzAB	6	1	5	0.25	0.60	0.25	0.60
21	1f3mAC	318	1	317	0.01	0.24	0.01	0.59
22	1f5qCD	1100	13	1087	0.03	0.21	0.04	0.33
23	1fbvAC	60	1	59	0.03	0.51	0.04	0.46
24	1finAB	1430	17	1413	0.14	0.35	0.03	0.40
25	1foeAB	185	3	182	0.16	0.14	0.06	0.18
26	1fq1AB	550	4	546	0.01	0.36	0.01	0.73
27	1fqvOP	14	8	6	0.88	0.27	0.88	0.27
28	1fxtAB	70	1	69	0.14	0.09	0.50	0.01
29	1g0uBJ	103	34	69	0.34	0.54	0.51	0.43
30	1g0uHI	103	34	69	0.28	0.59	0.37	0.45
31	1g0uLM	103	34	69	0.32	0.50	0.51	0.40
32	1g3nAB	2310	6	2304	0.01	0.33	0.00	0.45
33	1g3nEG	1210	14	1196	0.09	0.17	0.01	0.59
34	1g65DE	103	34	69	0.36	0.46	0.35	0.50
35	1g65IJ	103	34	69	0.32	0.53	0.36	0.47
36	1g65IZ	103	34	69	0.41	0.47	0.43	0.40
37	1g65KW	103	34	69	0.41	0.44	0.32	0.51

Query #	PDB ID	No. of Candidates	No. of Positives	No. of Negatives	AP (specific)	FP (specific)	AP (general)	FP (general)
38	1g65KX	103	34	69	0.48	0.36	0.37	0.46
39	1g65OP	103	34	69	0.38	0.50	0.36	0.54
40	1g65UV	103	34	69	0.29	0.56	0.55	0.34
41	1gl2AB	30	5	25	0.46	0.33	0.52	0.20
42	1gl2BC	6	4	2	1.00	0.00	0.82	0.50
43	1gotAB	1030	2	1028	0.25	0.47	0.00	0.40
44	1grnAB	374	6	368	0.17	0.10	0.03	0.36
45	1gw5AM	198	10	188	0.07	0.41	0.08	0.46
46	1gw5AS	110	5	105	0.08	0.42	0.12	0.31
47	1gw5BM	198	10	188	0.24	0.33	0.21	0.24
48	1gw5BS	110	5	105	0.21	0.31	0.16	0.40
49	1gw5MS	35	4	31	0.44	0.31	0.29	0.22
50	1h2tCZ	40	4	36	0.35	0.42	0.17	0.39
51	1h8eDG	4	2	2	0.75	0.50	0.75	0.50
52	1hq3AG	9	1	8	0.25	0.38	0.11	1.00
53	1hq3FH	4	1	3	0.25	1.00	0.25	1.00
54	1hr6EF	34	2	32	0.61	0.11	0.39	0.09
55	1i2mAB	148	1	147	0.50	0.01	0.14	0.04
56	1i50AB	154	2	152	0.42	0.02	0.29	0.02
57	1i50AK	77	2	75	0.58	0.01	0.58	0.01
58	1i50BI	6	2	4	0.50	0.38	0.75	0.25
59	1i7qAB	18	1	17	0.50	0.06	0.14	0.35
60	1ibrCD	666	7	659	0.40	0.06	0.13	0.16
61	1iruAG	105	34	71	0.44	0.42	0.39	0.48
62	1iruBC	105	34	71	0.38	0.48	0.38	0.50
63	1iruCD	105	34	71	0.38	0.46	0.33	0.52
64	1iruDE	105	34	71	0.40	0.43	0.36	0.48
65	1iruFG	104	34	70	0.36	0.49	0.35	0.50
66	1iruFN	104	34	70	0.39	0.42	0.48	0.36
67	1iruH2	105	34	71	0.36	0.49	0.31	0.55
68	1iruI1	105	34	71	0.27	0.61	0.29	0.56
69	1iruJK	105	34	71	0.58	0.27	0.33	0.48
70	1iruJ1	105	34	71	0.34	0.51	0.39	0.41
71	1iruKL	105	34	71	0.42	0.45	0.39	0.44
72	1iruLM	105	34	71	0.32	0.54	0.40	0.44
73	1iruNW	105	34	71	0.37	0.46	0.39	0.44
74	1iruOP	105	34	71	0.37	0.51	0.35	0.52
75	1iruS1	105	34	71	0.30	0.53	0.40	0.49
76	1iru12	105	34	71	0.27	0.60	0.33	0.48
77	1iw7CD	9	3	6	0.67	0.39	0.61	0.44

Query #	PDB ID	No. of Candidates	No. of Positives	No. of Negatives	AP (specific)	FP (specific)	AP (general)	FP (general)
78	1j2qDL	105	34	71	0.42	0.39	0.40	0.44
79	1j7dAB	119	4	115	0.28	0.54	0.27	0.59
80	1jatAB	116	4	112	0.04	0.55	0.03	0.72
81	1jfiAB	12	2	10	0.70	0.15	0.42	0.20
82	1jm7AB	11	2	9	0.63	0.33	0.37	0.28
83	1jr3BE	456	23	433	0.08	0.47	0.25	0.28
84	1jr3CD	31	5	26	0.41	0.32	0.48	0.18
85	1k5dDF	555	4	551	0.57	0.16	0.24	0.17
86	1k5dJK	111	3	108	0.32	0.06	0.56	0.02
87	1k83AI	231	3	228	0.51	0.01	0.47	0.01
88	1k8kAB	65	3	62	0.49	0.13	0.11	0.37
89	1k8kAD	11	2	9	0.58	0.11	0.25	0.50
90	1k8kAE	10	2	8	0.58	0.13	0.64	0.31
91	1k8kBF	10	2	8	0.83	0.06	0.58	0.13
92	1k8kBG	10	2	8	0.83	0.06	1.00	0.00
93	1k8kCF	81	1	80	1.00	0.00	1.00	0.00
94	1keeGH	80	1	79	0.25	0.04	0.33	0.03
95	1kfuLS	24	1	23	1.00	0.00	1.00	0.00
96	1ki1AB	180	3	177	0.24	0.05	0.09	0.16
97	1kx5EF	6	1	5	0.25	0.60	0.25	0.60
98	1kyoAB	34	2	32	0.45	0.06	0.24	0.14
99	1l4aBD	6	1	5	0.33	0.40	0.17	1.00
100	1lb1CD	148	3	145	0.18	0.08	0.08	0.16
101	1ldjAB	44	5	39	0.94	0.01	0.86	0.03
102	1ltxAR	6	1	5	1.00	0.00	0.50	0.20
103	1m1jEF	49	3	46	0.07	0.55	0.05	0.85
104	1m2vAB	13	3	10	0.25	0.60	0.37	0.33
105	1n1jAB	20	2	18	0.63	0.17	0.38	0.19
106	1n4pCD	6	3	3	0.64	0.33	0.81	0.22
107	1ni4AD	13	1	12	0.13	0.58	0.10	0.75
108	1nt2AB	3	2	1	0.83	0.50	0.83	0.50
109	1nvwRS	148	2	146	0.38	0.02	0.04	0.24
110	1oe9AB	35	8	27	0.83	0.13	0.53	0.19
111	1ofhCI	50	5	45	0.22	0.33	0.10	0.68
112	1p22AB	2	1	1	1.00	0.00	1.00	0.00
113	1pp9AB	36	2	34	0.38	0.10	0.18	0.19
114	1q5qAI	69	19	50	0.48	0.26	0.34	0.43
115	1qbkBC	1110	9	1101	0.20	0.08	0.02	0.23
116	1qdlAB	16	1	15	1.00	0.00	0.33	0.13
117	1qgkAB	23	2	21	0.58	0.26	0.70	0.07

Query #	PDB ID	No. of Candidates	No. of Positives	No. of Negatives	AP (specific)	FP (specific)	AP (general)	FP (general)
118	1qs0AB	13	1	12	0.09	0.83	0.14	0.50
119	1qviAY	35	8	27	0.49	0.19	0.32	0.37
120	1r4mHL	56	1	55	0.14	0.11	0.14	0.11
121	1rypBI	103	34	69	0.33	0.51	0.33	0.63
122	1rypCD	103	34	69	0.39	0.46	0.41	0.52
123	1rypFN	103	34	69	0.45	0.41	0.40	0.42
124	1rypHI	103	34	69	0.35	0.59	0.38	0.45
125	1rypH2	103	34	69	0.40	0.42	0.37	0.50
126	1rypI1	103	34	69	0.32	0.52	0.38	0.49
127	1rypI2	103	34	69	0.57	0.33	0.38	0.39
128	1rypLM	103	34	69	0.33	0.50	0.36	0.44
129	1rypS1	103	34	69	0.59	0.32	0.55	0.35
130	1s3sFG	4	1	3	1.00	0.00	1.00	0.00
131	1s63AB	6	3	3	0.81	0.22	0.83	0.33
132	1sfcAD	10	3	7	0.57	0.43	0.39	0.52
133	1sfcBC	6	2	4	0.83	0.13	1.00	0.00
134	1sfcEF	30	5	25	0.19	0.58	0.24	0.41
135	1sxjAB	850	38	812	0.31	0.36	0.28	0.30
136	1sxjAE	31	1	30	0.33	0.07	0.50	0.03
137	1sxjBC	813	38	775	0.36	0.34	0.26	0.34
138	1sxjCD	811	40	771	0.30	0.34	0.32	0.35
139	1sxjDE	688	31	657	0.28	0.31	0.30	0.26
140	1t2kCD	9	1	8	0.25	0.38	0.20	0.50
141	1tafAB	2	1	1	1.00	0.00	1.00	0.00
142	1tcoAC	52	2	50	0.06	0.45	0.05	0.59
143	1tt5AB	44	4	40	0.42	0.49	0.36	0.50
144	1tvkAB	15	1	14	0.20	0.29	0.50	0.07
145	1u7eAB	216	5	211	0.32	0.15	0.19	0.11
146	1ukvGY	34	3	31	0.59	0.09	0.83	0.03
147	1umcCD	13	1	12	0.09	0.83	0.08	1.00
148	1ur6AB	30	1	29	0.10	0.31	0.09	0.34
149	1v11AB	13	1	12	0.09	0.83	0.11	0.67
150	1vg0AB	70	13	57	0.87	0.04	0.55	0.13
151	1vrqAB	64	1	63	0.02	0.98	0.02	1.00
152	1w0jCD	10	3	7	0.58	0.43	0.67	0.33
153	1w85CD	13	1	12	0.08	0.92	0.09	0.83
154	1w98AB	1199	17	1182	0.21	0.17	0.05	0.35
155	1wa5AC	370	7	363	0.03	0.39	0.02	0.50
156	1wa5BC	184	1	183	0.02	0.33	0.05	0.11
157	1wq1RG	148	2	146	0.01	0.82	0.01	0.82

Query #	PDB ID	No. of Candidates	No. of Positives	No. of Negatives	AP (specific)	FP (specific)	AP (general)	FP (general)
158	1xcgAB	180	3	177	0.17	0.06	0.04	0.30
159	1xewXY	133	6	127	0.59	0.02	0.64	0.02
160	1xo2AB	1320	17	1303	0.26	0.22	0.02	0.45
161	1y56AB	90	1	89	0.04	0.28	0.01	1.00
162	1y8qCD	45	4	41	0.33	0.54	0.14	0.70
163	1y8rBC	18	2	16	0.64	0.16	0.36	0.25
164	1ya7CJ	105	34	71	0.42	0.41	0.34	0.52
165	1z2cAB	76	3	73	0.72	0.02	0.15	0.42
166	1z5sAB	30	1	29	0.11	0.28	0.25	0.10
167	2b4sAB	452	5	447	0.05	0.15	0.03	0.19
168	2ba0AD	10	2	8	0.35	0.56	0.23	0.69
169	2ba0FI	19	4	15	0.60	0.22	0.52	0.22
170	2ba1AD	15	3	12	0.64	0.28	0.74	0.31
171	2ba1BG	15	4	11	0.36	0.45	0.26	0.70
172	2bcjAQ	1080	5	1075	0.01	0.54	0.00	1.00
173	2bkiAB	45	8	37	0.52	0.22	0.38	0.38
174	2bkuAB	888	6	882	0.08	0.15	0.13	0.20
175	2bl0AB	8	2	6	0.42	0.42	0.29	0.58
176	2bl0AC	9	2	7	0.33	0.43	0.27	0.57
177	2br2EF	19	4	15	0.51	0.32	0.29	0.40
178	2btfAP	11	1	10	1.00	0.00	0.50	0.10
179	2bykCD	16	2	14	0.63	0.21	0.39	0.21
180	2c35EF	2	1	1	1.00	0.00	1.00	0.00
181	2ey4AE	2	1	1	1.00	0.00	1.00	0.00
182	3gtuAB	9	1	8	0.14	0.75	0.13	0.88

Supplementary Table S4 - **The 14 protein pair candidates of 1a2kAD to search yeast proteome.**

Homologs of 1a2kA	Homologs of 1a2kD	Exp[a]	SP energy[b]	SP energy (normal)	GE energy[c]	IDE1[d]	IDE[e]	Function1[f]	Function 2[g]
NTF2	GSP1	*P*	**68.4**	**0.5**	20.7	43	80	Nuclear envelope protein in nucleocytoplasmic transport	GTP binding protein involve in nuclear organization
NTF2	GSP2	*P*	**68.4**	**0.5**	20.7	43	79	Nuclear envelope protein in nucleocytoplasmic transport	GTP binding protein involve in nuclear organization
BRE5	YPT6	*N*	15.6	0.11	**-31.2**	21	26	Ubiquitin protease cofactor, coregulate vesicle transport	GTPase, involved in the secretory pathway
BRE5	GSP1	*N*	11	0.08	**-22.2**	21	80	Ubiquitin protease cofactor, coregulate vesicle transport	GTP binding protein involve in nuclear organization
BRE5	GSP2	*N*	11	0.08	**-22.2**	21	79	Ubiquitin protease cofactor, coregulate vesicle transport	GTP binding protein involve in nuclear organization
BRE5	YPT7	*N*	10.6	0.08	**-24**	21	24	Ubiquitin protease cofactor, coregulate vesicle transport	GTPase, required for homotypic fusion event
BRE5	RHO3	*N*	7.7	0.06	-18.6	21	24	Ubiquitin protease cofactor, coregulate vesicle transport	Non-essential small GTPase involved in the establishment of cell polarity.
BRE5	RHO2	*N*	6.7	0.05	-17.2	21	25	Ubiquitin protease cofactor, coregulate vesicle transport	Non-essential small GTPase of involved in microtubule assembly
BRE5	YPT31	*N*	5.7	0.04	-18.8	21	25	Ubiquitin protease cofactor, coregulate vesicle transport	GTPase, involved in the exocytic pathway;
BRE5	YPT11	*N*	4.4	0.03	**-23.4**	21	15	Ubiquitin protease cofactor, coregulate vesicle transport	Rab-type small GTPase mediate distribution of mitochondria to daughter cells
BRE5	TEM1	*N*	4.2	0.03	**-27**	21	21	Ubiquitin protease cofactor, coregulate vesicle transport	GTP-binding protein involved in termination of M-phase
BRE5	VPS21	*N*	2.7	0.02	**-20.7**	21	27	Ubiquitin protease cofactor, coregulate vesicle transport	GTPase required for transport during endocytosis
BRE5	SAR1	*N*	-23.2	-0.17	-7	21	20	Ubiquitin protease cofactor, coregulate vesicle transport	GTPase, component of COPII coat of vesicles
BRE5	MSS1	*N*	-35.2	-0.25	3.9	21	15	Ubiquitin protease cofactor, coregulate vesicle transport	Mitochondrial protein, modify the wobble uridine

[a] Exp means the functional annotations for the candidates, ***P*** represent known interacting proteins interaction and ***N*** represent the non-interacting proteins defined by Jasen et al.
[b] SP energy is the abbreviation of the "specific interfacial energy" which is calculated from *pair*PSSM of 1a2kAD.
[c] GE energy is the abbreviation of the "general interfacial energy" which is calculated from general empirical matrix.
[d] IDE1 means the sequence identity percentage between the candidate protein and 1a2k A chain.
[e] IDE2 means the sequence identity percentage between the candidate protein and 1a2k D chain.
[f] The functional annotation for the protein of candidate homologous to 1a2k A chain.
[g] The functional annotation for the protein of candidate homologous to 1a2k D chain.

Supplementary Table S5 - Statistic of homologous PPIs identified by *pair*PSSM for seven organisms commonly used in molecular research projects.

**Species**	**Number of proteins**	**Number of homologous PPIs (*pair*PSSM)**	**Number of PPIs (DIP)**
Homo sapiens (Human)	56924	223151	12975
Mus musculus (house mouse)	29571	112114	1233
Rattus norvegicus (Norway rat)	24115	71407	520
Caenorhabditis elegans (nematode)	22729	17242	5107
Drosophila melanogaster (fruit fly)	19620	41665	23446
Saccharomyces cerevisiae (baker's yeast)	5877	1850	25165
Escherichia coli	4850	477	12785

Supplementary Table S6- The result of 1evtBD to model seven FGF/receptor complexes.

Homologs of 1evtB	Homologs of 1evtD	Binding affinity[a]	SP energy[b]	SP energy (normal)	GE energy[c]	IDE1[d]	IDE2[e]
FGF4	FGFR2c	**94.3**	**105.7**	**0.92**	-1.6	35.8	76.3
FGF4	FGFR3c	**69.1**	**104.8**	**0.91**	-1	35.8	73
FGF4	FGFR4	**108**	**103.4**	**0.9**	0.5	35.8	66
FGF4	FGFR1c	**102.3**	**102.5**	**0.89**	0.5	35.8	99.1
FGF4	FGFR2b	**14.9**	**101.2**	**0.88**	-1.9	35.8	69.3
FGF4	FGFR1b	**15.6**	**98**	**0.85**	-0.4	35.8	86
FGF4	FGFR3b	1	**97.3**	**0.84**	1.3	35.8	61.9

[a] Binding affinity of each FGF/receptor complex is recoded in Ornitz's study.
[b] SP energy is the abbreviation of the "specific interfacial energy" which is calculated from pairPSSM of 1evtBD.
[c] GE energy is the abbreviation of the "general interfacial energy" which is calculated from general empirical matrix.
[d] IDE1 means the sequence identity percentage between the candidate protein and 1evt B chain.
[e] IDE2 means the sequence identity percentage between the candidate protein and 1evt D chain.
